# Associations between biomarkers and clinical outcome in people hospitalized with HIV: a retrospective cohort

**DOI:** 10.1590/1518-8345.7473.4952

**Published:** 2026-10-07

**Authors:** Jarbas da Silva Ziani, Jenifer Härter, Bibiana Letícia Nogara, Francielle Liz Monteiro, Laís Mara Caetano da Silva Corcini, Cláudia Zamberlan

**Affiliations:** 1 Universidade Federal de Santa Maria, Santa Maria, RS, Brazil.; 2 Universidade Federal do Pampa, Departamento de Enfermagem, Uruguaiana, RS, Brazil.; 3 Universidade Franciscana, Departamento de Biomedicina, Santa Maria, RS, Brazil.; 4 Universidade Federal de Santa Maria, Centro de Ciências da Saúde, Santa Maria, RS, Brazil.; 5 Universidade Franciscana, Departamento de Enfermagem, Santa Maria, RS, Brazil.

**Keywords:** HIV, Biomarkers, Prognosis, Patient Acuity, Risk Factors, Nursing

## Abstract

**Objective:**

to verify the associations between biomarkers and their relationship with the clinical outcome of people hospitalized with HIV infection.

**Method:**

retrospective cohort of documentary character. People hospitalized with a diagnosis of HIV infection were included, with hospital discharge and death as outcomes. Sociodemographic, clinical, and biomarker variables were evaluated using Fisher’s exact analysis, Pearson’s chi-square, and Cox regression.

**Results:**

among the 102 participants analyzed, 27 (26.5%) died during hospitalization. Among the factors associated with the risk of death, viral load greater than 1000 copies/mL (HR: 3.99; p = <0.023), CD4+ T lymphocyte count up to 200 copies/mm³ (HR: 1.92; p = <0.035), glucose greater than 126 mg/dL (HR: 5.40; p = <0.017), creatinine greater than 1.20 mg/dL (HR: 9.75; p = <0.012) and triglycerides above 201 mg/dL (HR: 11.75; p = <0.000) stand out.

**Conclusion:**

it was found that immunological, metabolic, renal and lipid biomarkers play an essential role in the prognostic evaluation of people hospitalized with HIV infection, being strongly associated with the risk of death.

## Highlights

**(1)** A mortality rate of 26.5% (n=27) was identified during the study.

**(2)** There was an association with death among people with a viral load above 1000 copies/mL.

**(3)** An association was observed between death and LT-CD4+ levels lower than 200 cells/mL.

**(4)** People with creatinine greater than 1.20 mg/dL had a 9.75 times higher risk.

**(5)** Triglycerides higher than 201 mg/dL represented the highest risk of death.

## Introduction

Globally, there are about 39.9 million people living with the Human Immunodeficiency Virus (HIV). Of these, 1.3 million were new infections, and 630 thousand people died due to Acquired Immunodeficiency Syndrome (AIDS), according to 2023 indicators. When analyzing the HIV panorama in Brazil, it is observed that, since 2015, the epidemic has been under control. However, the Joint United Nations Programme on HIV/AIDS (UNAIDS) has confirmed that rates in adults have increased again since 2000, compared to the years 1990 to 2022^1^.

To increase the quality and longevity of people living with HIV, Antiretroviral Therapy (ART) was developed, which acts to suppress viral replication and, consequently, significantly reduces morbidity and mortality resulting from immunosuppression caused by HIV. This advance allowed these people to have a life expectancy similar to that of the population without the virus^2^. In addition, ART altered the course of the disease, transforming it into a controllable chronic condition, in which non-infectious complications became more frequent^1^ ^)-(^ ^3^.

Considering these changes and the risk factors for comorbidities in people living with HIV, global projections are worrying. It is estimated that, by 2030, more than 70% of these people worldwide will be over 50 years old^4^. This indicates that this population is aging and, therefore, presents a high prevalence of metabolic diseases, including increased adiposity, insulin resistance, diabetes and dyslipidemias, which leads to an increase in cases of Chronic Vascular Diseases (CVD), in addition to kidney, liver, metabolic and multimorbidity complications^4^ ^)-(^ ^7^.

In view of these aspects, the hospitalization of people living with HIV has become a global challenge for the health system, especially in low- and middle-income countries, where access to health services is still limited. This often leads these people to serious complications, such as opportunistic infections and severe immunosuppression^1^ ^),(^ ^8^ ^)-(^ ^9^. The lack of early diagnosis, the difficulty in accessing ART and the interruption of treatment increase the vulnerability of these people, resulting in frequent hospitalizations^10^.

In addition, the multicomorbidities affecting people living with HIV have increased the demand for specialized medical services, as well as increased treatment costs and overload on hospitals. This makes it essential to monitor biomarkers in this population, as they play a key role in prognostic assessment. Inflammatory, immunological, metabolic, renal and lipid biomarkers can provide essential information on disease severity, response to treatment and clinical progression, helping to identify early those at higher risk of death^11^ ^)-(^ ^14^.

In view of a survey carried out in the literature, a lack of studies on the association between biomarkers and clinical outcomes in people hospitalized due to HIV was identified, an instigating aspect, given that this theme is included in the national agenda of health research priorities in Brazil^15^ ^)-(^ ^16^. The originality of this study lies in the innovative approach of exploring biochemical variations in hospitalized people, seeking to establish possible relationships between biomarkers and clinical prognosis. 

Furthermore, this research is justified by the relevance of recognizing and identifying the determining factors in the evolution of AIDS in people living with HIV, contributing to a better understanding of the biological mechanisms involved in the progression of the disease. Based on the findings, it will be possible to outline effective strategies for clinical management, optimize clinical decision-making, and strengthen evidence-based practice.

In this way, this study not only improves scientific knowledge about this health condition, but also generates direct impacts on the quality of care and outcomes of people hospitalized for HIV. Thus, the aim of this study is to verify the associations between biomarkers and their relationship with the clinical outcome of hospitalized people with HIV infection.

## Method

### Study design and setting

This is a retrospective, documental cohort study, guided by the guidelines of the Strengthening the Reporting of Observational Studies in Epidemiology (STROBE)^17^.

The study was conducted in the tertiary care network, in a clinical hospitalization unit of a medium-complexity hospital located in a large municipality in Rio Grande do Sul-RS, Brazil. The choice of the location is due to the fact that it is a reference for the care of people living with HIV. In addition, the hospital has a fruitful insertion in the local and regional health scenario, characterizing itself as a teaching hospital that provides care exclusively through the Unified Health System (SUS).

The hospital is characterized by its technical quality and has 112 inpatient beds, distributed among the clinical, surgical, maternity, pediatric and psychiatric hospitalization units. In this context, it is noteworthy that, for the research, only the medical records of the clinical hospitalization ward were analyzed, which has 34 beds, 15 male, 15 female and four for isolation.

In addition, the municipality of the study is located in the central region of the state and has an estimated population of 271,633 inhabitants in 2023. The city is composed of a district headquarters, which covers eight administrative regions and a total of 42 neighborhoods, in addition to nine districts. With regard to the health network, the organization occurs in three gradual levels of care, within the scope of the SUS^18^. Furthermore, the investigation of the scenario becomes fundamental, considering the high rates of HIV in the municipality. In 2022, it was estimated that approximately 2.5 thousand people were living with HIV in this municipality, of which 157 corresponded to new cases and about 800 had abandoned treatment. This scenario contributes to the increase in hospitalizations due to the progression of AIDS and its clinical complications^18^.

### Study population and period

All electronic medical records of people hospitalized with HIV diagnosis from January 1, 2022 to January 31, 2023 were analyzed. This time frame was chosen due to the fact that, in 2020 and 2021, 90% of the beds in the unit studied were intended for the care of people with COVID-19. In this way, we sought to minimize possible interference of the pandemic in the data analysis.

### Selection criteria

The study population was composed of people over 18 years of age, whose hospitalization in the clinical unit was at least 72 hours. Regarding the exclusion criteria, medical records that presented more than 80% of the data were illegible and/or incomplete for the variables analyzed in the study.

To identify the eligible medical records, the researchers analyzed all available medical records from that period, referring to people admitted to the unit in question, and selected only those whose hospitalization was related to HIV. In addition, to minimize selection bias, the medical records were analyzed by double checking, carried out by independent researchers.

### Instruments used for information collection

Data were collected from an instrument developed specifically for the present study, considering the recommendations of the clinical protocol and therapeutic guidelines for the management of HIV infection in adults of the Ministry of Health (MS)^19^. In addition, the instrument was appreciated by five professionals experienced in the theme of HIV for the purpose of content and semantic analysis. The specialists received the instrument by email with a deadline of up to 20 days to return with their considerations. The aspects evaluated were: being easy to understand, clarity in the writing, relevance of the items, presence of ambiguities. Also, the document contained a space for suggestions.

After this phase, a pilot test was carried out with three medical records eligible for the study, which proved to be adequate for the research. It is noteworthy that the medical records of the pilot test were not considered for the analysis of the study.

### Study variables

For the analysis of the biochemical variables, the predicted values expressed in the laboratory tests were initially collected. Subsequently, these values were categorized. Demographic characteristics were collected regarding the variables analyzed: gender (male/female), age in complete years, diagnosis of Diabetes Mellitus (DM) (yes/no), Chronic Kidney Disease (CKD) (yes/no), Systemic Arterial Hypertension (SAH) (yes/no), Tuberculosis/HIV co-infection (TB/HIV) (yes/no), other comorbidities (yes/no), and outcome (death/hospital discharge). For the regression analysis, the raw values expressed in the participants’ examinations (without categorization) were used.

The hematological biochemical markers used and their respective parameters were as follows: viral load (Less than 1000 copies/mL/Equal to or greater than 1000 copies/mL), CD4+ T lymphocytes (Less than 200 copies/cubic millimeter (mm^3^)/Equal to or greater than 200 copies/mm^3^); erythrocytes (4.5 to 6.1 mm^3^/less than 4.49 mm^3^), hemoglobin (less than 12.79 milligram (mg)/deciliter (dL)/12.8 to 17.8 mg/dL/upper 17.9 mg/dL), hematocrit (lower than 38.9 mg/dL/39 to 53 mg/dL/upper 53.1 mg/dL), leukocytes (lower than 3599 mm^3^/3600 to 11000 mm^3^/upper 11001 mm^3^), platelets (lower than 149,999 mm^3^/150,000 to 450,000 mm^3^/upper 450,001 mm^3^) and blood glucose (lower than 125 mg/dL and higher than 126 mg/dL). Total cholesterol (TC) (up to 190 mg/dL/greater than 191 mg/dL), lipoproteins responsible for cholesterol transport. High-Density Lipoproteins (HDL) (less than 39.1 mg/dL/40 to 60 mg/dL), Low-Density Lipoprotein (LDL) (100 to 159 mg/dL/Greater than 160 mg/dL), triglycerides (TG) (150 to 200 mg/dL/Greater than 201 mg/dL).

The biochemical markers of electrolytes and renal function were urea (19.0 to 43.0 mg/dL/greater than 43.1 mg/dL), creatinine (0.70 to 1.19 mg/dL/greater than 1.20 mg/dL), sodium (137.0 to 145.9 mmol/L/greater than 146 mmol/L), potassium (3.5 to 5.1 mmol/L/greater than 5.2 mmol/L). Biochemical markers of liver function: aspartate aminotransferase (AST) (up to 40.9 U/L/greater than 41 U/L), alanine aminotransferase (ALT) (up to 41 U/L/greater than 41.1 U/L), total bilirubin (BT) (0.3 to 1.20 mg/dL/greater than 1.21 mg/dL), direct bilirubin (BD) (up to 0.20 mg/dL/greater than 0.21), indirect bilirubin (BI) (up to 1.0 mg/dL/greater than 1.1 mg/dL).

### Data collection

Data collection was carried out from February to June 2023 by the study’s principal investigator and another assistant collector, both duly trained.

To control for biases, both in measurement and selection, the medical records were analyzed and collected by the two researchers and then transported to a database in Excel^®^ spreadsheets (Microsoft, version 10, USA). Thus, the data went through a process of double entry, followed by validation to promote its reliability. It is noteworthy that a third reviewer was not necessary for the analysis of the medical records.

### Data processing and analysis

Regarding data analysis, the Statistical Package for the Social Sciences (SPSS) software, version 24, was used. Descriptive statistical techniques and measures of association were applied. Continuous variables were presented by their measures of central tendency, namely, mean, standard deviation (SD) (±), minimum and maximum. Categorical variables were expressed as absolute (n) and relative (%) frequency distribution. In addition, to compare the groups according to the clinical outcome, i.e., death or hospital discharge, unpaired tests were used, in addition to Pearson’s chi-square test or Fisher’s exact test, considering the nature of continuous and categorical variables, respectively.

To determine the risk of death of the participants, the Cox proportional hazards model was used, which was used to recognize the covariates that influenced the time from the beginning of hospital admission to the outcome of the case. It is noteworthy that variables with a p-value ≤0.05 in the bivariate analysis were included in the multivariate analysis model. The global fit of the model was obtained by means of the likelihood ratio test. There was an association in relation to biochemical markers and the outcome (death and hospital discharge) and the covariates individually associated with the outcomes, with p<0.05.

Thus, the covariates were included in the Cox regression model for the analyses. It was established as a p-critical <0.05 in the multiple analysis to accept the association between the person’s outcome and biochemical alterations. The Hazard Ratio (HR) was calculated in the crude and adjusted analyses with a 95% confidence interval, and the quality of the fit was evaluated through the distribution of the residuals.

### Ethical aspects

The research was approved by the Research Ethics Committee (CEP) of the *Universidade Franciscana* (UFN) on 12/19/2022, under opinion No. 5,828,312.

## Results

During the study period, 1,328 people were admitted to the clinical unit, 117 of which were hospitalizations related to HIV infection. After applying the inclusion and exclusion criteria, 15 medical records were disregarded (10 for length of stay of less than 72 hours and five for presenting more than 80.0% of the data incomplete), resulting in 102 participants eligible for analysis.

Of the total sample, the mean age was 43.3 years (SD±12), with a minimum age of 18 and a maximum of 78 years. Regarding the clinical outcome, 27 (26.5%) cases died during the study, due to HIV, and 75 (73.5%) were discharged from the hospital. The mean length of hospital stay recorded in the participants’ medical records was 19 days, with a minimum period of five and a maximum of 45 days (SD±9).

Of the total, 60 (58.8%) were male and, in relation to the use of ART, 75 (73.5%) were under treatment during hospitalization and 27 (16.8%) had interrupted. In addition, the clinical characteristics and prevalence of comorbidities presented by the participants are detailed in Table 1 according to the clinical outcome.

**Table 1:** Sociodemographic and clinical characteristics of people hospitalized with HIV. Santa Maria, RS, Brazil, 2023

Variable	Clinical outcome				
	Hospital discharge		Death		
	n*	(%)^†^	n*	(%)^†^	p-value^‡^
Gender					**0.013** ^§^
Male	39	52.0%	22	81.5%	
Female	36	48.0%	5	18.5%	
Use of ART^||^					0.000^¶^
Yes	75	100%	-	-	
No	-	-	27	100%	
Diabetes Mellitus					**0.000** ^§^
Yes	22	29.3%	17	62.9%	
No	53	70.7%	10	37.1%	
Chronic kidney disease					**0.002** ^§^
Yes	69	92.0%	18	66.7%	
No	6	8.0%	9	33.3%	
Systemic arterial hypertension					**0.000** ^§^
Yes	25	33.3%	19	70.4%	
No	50	66.7%	8	29.6%	
TB/HIV co-infection**					0.357
Yes	23	30.7%	11	40.7%	
No	52	69.3	16	59.3%	
Other comorbidities					**0.028** ^¶^
Yes	25	33.3%	4	14.8%	
No	50	66.7%	23	85.2%	

*n = Sample size according to the outcome; ^†^% = Percentage; ^‡^p-value = Statistically significant value at the 5% level (p ≤ 0.05); ^§^Pearson’s chi-square test; ^||^ART = Antiretroviral Therapy; ^¶^Fisher’s Exact Test; **TB/HIV = Tuberculosis/Human Immunodeficiency Virus

Regarding the reason for the participants’ admission to the clinical hospitalization unit, the following were identified: immunosuppression - 30 people (29.4%), pneumonia - 10 (9.8%), tuberculosis - six (5.9%), opportunistic infection under investigation - four (3.9%), cryptococcosis - three (2.9%), malnutrition - three (2.9%), viral hepatitis - three (2.9%), herpes zoster - three (2.9%), pancreatitis - three (2.9%), Kaposi’s sarcoma - three (2.9%), sexual abuse - two (2.0%), oral and esophageal candidiasis - two (2.0%), neurotoxoplasmosis - two (2.0%), pneumocystosis - two (2.0%), sepsis - two (2.0%) and others - 24 (23.5%).

Table 2 presents and compares the differences between the hematological markers of the study participants and their relationship with clinical outcomes. A statistical association was observed between death and participants who had an erythrocyte count below 4.49/mm^3^, hemoglobin levels below 12.79 mg/dL, and platelets below 149,999 mm^3^, with a value of p=0.000. 

**Table 2:** Association of hematological biomarkers and lipid profile in people hospitalized with HIV, according to clinical outcome. Santa Maria, RS, Brazil, 2023

Variable	Clinical outcome				
	Hospital discharge		Death		
	n*	(%)^†^	n*	(%)^†^	p-value^‡^
Erythrocytes (mm^3§^)					**0.000** ^||^
4.5 to 6.1	51	50.0%	3	2.9%	
Lower than 4.49	24	23.5%	24	23.6%	
Haemoglobin (mg/dL^¶^)					0.000^||^
Lower than 12.79	21	20.7%	25	24.5%	
12.8 to 17.8	54	52.9%	2	2.0%	
Hematocrit (mg/dL^¶^)					0.321**
Lower than 38.9	67	65.7%	22	21.6%	
39 to 53	8	7.8%	5	4.9%	
Leukocytes (mm^3§^)					0.836**
Lower than 3599	11	10.8%	4	3.9%	
3600 to 11000	46	45.2%	15	14.7%	
Greater than 11001	18	17.6%	8	7.8%	
Platelets (mm^3§^)					**0.000** ^||^
Lower than 149.999	11	10.8%	14	13.7%	
150.000 to 450.000	59	57.8%	10	9.8%	
Greater than 450.001	5	4.9%	3	2.9%	
Total Cholesterol (mg/dL^¶^)					**0.000** ^||^
Up to 190	53	52.0%	3	2.9%	
Greater than 191	22	21.6%	24	23.5%	
HDL^††^ (mg/dL^¶^)					**0.000** ^||^
Lower than 39.9	38	37.2%	26	25.5%	
40 to 60	37	36.3%	1	1.0%	
LDL^‡‡^ (mg/dL^¶^)					**0.000** ^||^
100 to 159	47	46,1%	3	2,9%	
Greater than 160	28	27.5%	24	23.5%	
Triglycerides (mg/dL^¶^)					**0.000** ^||^
150 to 200	64	61.7%	1	1.0%	
Greater than 201	11	10.8%	27	26.5%	

*n = Sample size according to the outcome; ^†^% = Percentage; ^‡^p-valor = Statistically significant value at the 5% level (p ≤ 0,05); ^§^mm^3^ = Cubic millimeters; ^||^Fisher’s Exact Test; ^¶^mg/dL = Milligrams per deciliter; **Pearson’s chi-square test; ^††^HDL = High-density lipoproteins; ^‡‡^LDL = Low-density lipoprotein

In addition, an association was found between lipid profile and clinical outcome. Participants with total cholesterol above 191 mg/dL, HDL lower than 39.9 mg/dL, LDL higher than 160 mg/dL, and triglycerides higher than 201 mg/dL showed a greater association with death (p=0.000).

Regarding the participants’ renal function markers, it was identified that there is a statistical association between the outcome of death and the markers of urea, creatinine, sodium, and potassium, as highlighted in Table 3.

**Table 3:** Association of renal biomarkers in people hospitalized with HIV, according to clinical outcome. Santa Maria, RS, Brazil, 2023

Variable	Clinical outcome				
	Hospital discharge		Death		
	n*	(%)^†^	n*	(%)^†^	p-value^‡^
Urea (mg/dL^§^)					**0.000** ^||^
9.0 to 43.0	8	7.8%	20	19.6%	
Greater than 43.1	67	65.7%	7	6.9%	
Creatinina (mg/dL^§^)					**0.000** ^¶^
0.70 to 1.19	62	60.8%	4	3.9%	
Greater than 1.20	13	12.7%	23	22.6%	
Sodium (mmol/L**)					**0.000** ^||^
137.0 to 145.9	69	67.6%	10	9.8%	
Greater than 146	6	5.9%	17	16.7%	
Potassium (mmol/L^**^)					**0.000** ^¶^
3.5 to 5.1	71	69.6%	6	5.9%	
Greater than 5.2	4	3.9%	21	20.6%	

*n = Sample size according to the outcome; ^†^% = Percentage; ^‡^p-value = Statistically significant value at the 5% level (p ≤ 0.05); ^§^mg/dL = Milligrams per deciliter; ^||^Pearson’s chi-square test; ^¶^Fisher’s Exact Test; **mmol/L = Millimol per liter

As shown in Table 4, the evolution to death showed a significant association with liver markers. The death group had a higher prevalence of results that indicate a worse prognosis, specifically for AST (p=0.000), ALT (p=0.001), TB (p=0.003) and DB (p=0.032).

**Table 4:** Association of hepatic biomarkers in people hospitalized with HIV, according to clinical outcome. Santa Maria, RS, Brazil, 2023

Variable	Clinical outcome				
	Hospital discharge		Death		
	n*	(%)^†^	N*	(%)^†^	p-value^‡^
AST^§^ (U/L^||^)					**0.000** ^¶^
up to 40.9	47	46.1%	4	3.9%	
Greater than 41	28	27.5%	23	22.5%	
ALT** (U/L^||^)					**0.001** ^††^
Up to 41	60	58.8%	10	9.8%	
Greater than 41.1	15	14.7%	17	16.7%	
Total bilirrubin (mg/dL^‡‡^)					**0.003** ^††^
0.3 to 1.20	41	40.2%	5	4.9%	
Greater than 1.21	34	33.3%	22	21.6%	
Direct bilirrubin (mg/dL^‡‡^)					0.052^††^
Up to 0.20	45	44.1%	12	11.8%	
Greater than 0.21	30	29.4%	15	14.7%	
Indirect bilirrubin (mg/dL^‡‡^)					**0.032** ^¶^
Up to 1.0	22	21.6%	2	2.0%	
Greater than 1.1	53	52.0%	25	24.5%	

*n = Sample size according to the outcome; ^†^% = Percentage; ^‡^p-value = Statistically significant value at the 5% level (p ≤ 0.05); ^§^AST = Aspartate aminotransferase; ^||^U/L = Units per liter; ^¶^Fisher’s Exact Test; **ALT = Alanine aminotransferase; ^††^Pearson’s chi-square test; ^‡‡^mg/dL = Milligrams per deciliter

According to the data expressed in Table 5, the adjusted model identified an association with death among people who had a VC equal to or greater than 1000 copies/mL (p=0.023), CD4 T-lymphocytes equal to or less than 200 cells/mm^3^ (p=0.035), blood glucose equal to or greater than 126 mg/dL (p=0.017), creatinine equal to or greater than 1.20 mg/dL (p=0.012) and triglycerides equal to or greater than 201 mg/dL (p=0.000).

**Table 5:** Crude and adjusted Cox regression analysis for the risk of death in people hospitalized with HIV. Santa Maria, RS, Brazil, 2023

Variable	Crude HR* (CI 95%^†^)	p^‡^	Adjusted HR* (CI 95%^†^)	p^‡^
Viral load (copies/mL)				
Equal to or greater than 1000	9.78 (3.23-12.43)	0.000	3.99 (1.25-12.85)	**0.023** ^§^
CD4+ T L^||^ (copies/mm^3¶^)				
Equal to or lower than 200 cells/mm^3^	4.12 (1.78-2.12)	0.008	1.92 (1.85-2.36)	**0.035** ^§^
Glucose (mg/dL**)				
Greater than 126	4.49 (2.29-8.36)	0.005	5.40 (2.11-8.21)	**0.017** ^§^
Urea (mg/dL**)				
Greater than 43.1	0.16 (0.06-1.26)	0.007	0.19 (0.09-1.99)	0.195
Creatinine (mg/dL**)				
Greater than 1.20	10.12 (5.23-15.36)	0.009	9.75 (2.56-14.98)	**0.012** ^§^
Sodium (mmol/L^††^)				
Greater than 146	2.19 (1.34-3.60)	0.009	2.12 (1.27-3.51)	0.051
Potassium (mmol/L^††^)				
Greater than 5.2	3.25 (0.81-18.95)	0.007	5.23 (0.85-45.69)	0.089
Total Cholesterol (mg/dL**)				
Greater than 191	0.52 (0.08-2.36)	0.001	0.23 (02.3-2.15)	0.212
HDL^‡‡^ (mg/dL**)				
Less than 39.9	2.45 (0.88-6.33)	0.008	0.68 (0.23-1.65)	0.654
LDL^§§^ (mg/dL**)				
Greater than 160	2.45 (0.85-5.71)	0.004	1.63 (0.63-4.71)	0.854
Triglycerides (mg/dL**)				
Greater than 201	12.15 (2.75-15.63)	0.008	11.75 (4.18-23.26)	**0.000** ^§^

*HR = Hazard Ratio; ^†^CI = 95% confidence interval; ^‡^p = Level of significance; ^§^Statistically significant result; ^||^CD4+ T L= CD4+ T lymphocytes; ^¶^mm^3^ = Cubic millimeters; **mg/dL = Milligrams per deciliter; ^††^mmol/L = Millimol per liter; ^‡‡^HDL = High-density lipoproteins; ^§§^LDL = Low-density lipoprotein

In addition, it is observed that participants with VC results above 1000 copies/mL had approximately 3.99 times more risk of progressing to death, compared to those with results below 1000 copies/mL (adjusted HR: 3.99; 95% CI: 1.25-12.85). People who had a CD4+ T-lymphocyte count of less than 200 cells/mm^3^ had a 1.92 times higher risk of death compared to those who had a count of 200 mm^3^ or more (adjusted HR: 1.92; 95% CI: 1.85-2.36).

Participants who had a result greater than 126 mg/dL in the blood glucose biomarker had a 5.40 times higher risk of dying during hospitalization, when compared to people who had a value lower than 125 mg/dL (adjusted HR: 5.40; 95% CI: 2.11-8.21). It was observed that participants with a creatinine marker value greater than 1.20 mg/dL had a 9.75 times higher risk of death, compared to those with values lower than 1.19 mg/dL (adjusted HR: 9.75; 95% CI: 2.56-14-98).

In addition, participants who had triglyceride levels above 201 mg/dL had a risk of death 11.75 times higher than those who had lower values (adjusted HR: 11.75; 95% CI: 4.18-23.26).

## Discussion

In order to analyze the alterations between the biomarkers presented by people hospitalized with HIV infection, it is observed that VL values reinforce what has already been identified in the scientific literature. Studies indicate that people living with HIV using ART, with an undetectable viral load or between 200 and 1000 copies/mL, have better prognosis of infection^20^. On the other hand, hospitalized people with a VL greater than 1000 copies/mL are directly associated with advanced AIDS markers and have a higher risk of progression to death^21^.

In this context, the present study reinforces that people living with HIV who have CD4+ T lymphocyte counts lower than 200 copies/mm³ are more susceptible to opportunistic infections and, consequently, have a higher probability of progression to death due to the disease. A Brazilian study that analyzed the death profile of 109,636 people living with HIV from 2014 to 2019 identified results similar to the findings of the present study, showing that lower rates of CD4+ T lymphocytes were associated with mortality^22^. In addition, the results of this study demonstrate that people with CD4+ T lymphocyte counts lower than 200 copies/mm^3^ have approximately 1.92 times more risk of dying compared to those with values equal to or greater than 200 mm³ (p=0.035).

Similarly, a study carried out in Tanzania identified that people living with HIV have a risk of developing DM up to five times higher than those who do not live with the virus^23^. This association becomes even more significant when considering the findings of the present study, in which participants with blood glucose levels higher than 126 mg/dL had a 5.4 times higher risk of dying. It is noteworthy that the increase in blood glucose levels in people living with HIV can be explained, to a large extent, by insulin resistance induced by ART, a factor that contributes to the development of DM^23^.

In addition, it is noteworthy that soon after the introduction of ART, people living with HIV experience immune reconstitution, a process that can result in increased secretion of tumor necrosis factors alpha (TNF-α). This phenomenon can induce insulin resistance through several mechanisms that involve the insulin receptor signaling cascade^24^. In view of this, it is essential that health professionals maintain a continuous commitment to preventive strategies, including regular monitoring of glycemic levels. This approach aims to reduce the progression to DM, preventing the double burden of the disease in this population and, consequently, reducing the chances of a worse prognosis^23^.

The analysis of the association between renal markers and the outcome of death revealed a significant association between renal markers and the risk of death. People with values higher than 1.20 mg/dL had a 9.75 times higher risk of death. In view of this, it is essential to emphasize that renal impairment represents a serious problem in people living with HIV, being associated with several causes, including nephropathies resulting from HIV, nephrotoxicity due to medications, medical procedures and, especially, chronic kidney disease, which can be caused or aggravated by comorbidities^25^ ^)-(^ ^26^.

A study conducted in the United States indicated that people living with HIV have a risk of developing kidney failure up to 13 times higher than people who do not live with the virus, a value higher than that identified in the present study^27^. This divergence may be related to the free and universal access to treatment in Brazil, which may contribute to a better control of renal complications in this population.

In addition, the study suggests that all people diagnosed with HIV should be submitted to a detailed evaluation of renal alterations, considering the possibility of coexistence of multiple triggering factors^27^. This recommendation is in line with national protocols, which aim to ensure adequate monitoring of complications associated with HIV^28^.

Lipid profile markers proved to be relevant in the study, as they were associated with the outcome of death. In the United States, a study conducted with 981 people living with HIV identified similar results, showing that 41% of the participants had high levels of TC (p=0.000) and TG, while 21.4% of the people who died had an association with these markers (p=0.002)^4^. Thus, it is noted that the lipid profile found in this study presents values higher than those of the aforementioned study, but maintains the same significant association with death as an outcome (p=0.000).

A Brazilian study^22^ warns about the importance of health professionals considering strategies aimed at self-care and the adoption of healthy lifestyle habits, aiming to minimize these risk factors. Such factors are directly related to cardiovascular complications, which represent the main cause of morbidity and mortality among people living with HIV^29^.

In addition, the high prevalence of comorbidities in the sample of the present study, with 38.2% of DM diagnoses, 41.2% of SAH, and 85.3% of CKD, is in line with what has been observed globally in people living with HIV. The evolution in the prescription of medications for chronic diseases and the increasing prevalence of these comorbidities among these people, as demonstrated in a recent study in Belgium, underline the need for continuous monitoring and adequate management to avoid unfavorable outcomes^30^. The presence of these comorbidities, by itself, is already a factor of worse prognosis, and their interaction with the biochemical alterations discussed in this study may intensify the risk of death.

In view of the findings of this study, corroborated by the scientific literature^4^ ^),(^ ^29^, the importance of adherence to ART and the need to develop healthy lifestyle habits to minimize unfavorable clinical outcomes are highlighted. Although ART is associated with a higher risk of metabolic alterations and an increased probability of developing cardiovascular diseases in the long term, it remains the most effective strategy for the management of HIV infection, providing better prognosis and quality of life to people living with HIV^31^.

The prevalence of liver disease in people living with HIV varies between 4% and 18% in different regions of the world, and mortality rates related to liver damage are up to 10 times more frequent in this population compared to the general population^32^. A study carried out in Spain reaffirmed this finding, confirming that participants living with HIV and having liver impairment had a worse prognosis compared to those without HIV infection. The main causes of liver disease in this population included co-infections with Hepatitis C virus (HCV) and Hepatitis B virus (HBV), drug-induced liver injury, alcoholic disease, and nonalcoholic fatty liver disease^33^.

The authors also emphasize the importance of health professionals’ commitment, from the initial phase of the diagnosis of the infection, to the monitoring of liver and lipid markers, regardless of the combination of ART used^33^. This follow-up is essential to reduce the risk of liver disease progression and the development of hepatocellular carcinoma.

In view of the aspects highlighted, the relevance of clinical practice and future research on the subject is highlighted, as treatment interruption, recurrence and HCV co-infection in people living with HIV require rigorous follow-up, especially regarding residual fibrosis. In addition, well-designed studies that investigate the development of long-term metabolic disorders in co-infected people can significantly contribute to reducing the magnitude of this problem, helping in the development of more effective therapeutic strategies^33^.

The analysis of the participants’ reasons for hospitalization, which included opportunistic infections such as pneumonia (9.9%), tuberculosis (5.9%), cryptococcosis (2.9%), and sepsis (2.0%), reinforces the importance of attention to severe immunosuppression. The diversity of these clinical conditions underlines the vulnerability of this population to severe complications, which may explain the strong association between altered biomarkers and the outcome of death^8^ ^)-(^ ^9^.

In addition to the analysis of the markers that remained in the regression model, this study also investigated the relevance of hematological biomarkers. Although they did not have the statistical power to remain in the model, these biomarkers are of great importance in the immunity of people living with HIV. The analysis of the results of the participants’ blood count tests revealed a significant association between the presence of anemia and the progression to death p=0.000. This finding reinforces the results of a meta-analysis, which identified the presence of anemia in 78.8% of people living with HIV, associating this condition with worse health prognosis^34^.

Thus, the importance of performing periodic examinations in people living with HIV is highlighted, as well as the attention of health professionals to its alterations, since anemia has been cited as a marker of worse prognosis of infection and is mainly associated with reduced survival in these people^34^. It is worth noting that several factors can influence the development of anemia in people living with HIV, including the use of ART, the presence of inflammatory mediators, and the co-infections associated with the infection. These factors can compromise the proliferation and differentiation of hematopoietic stem/progenitor cells during hematopoiesis, culminating in the clinical picture of anemia^35^.

Regarding the limitations of the study, the use of secondary sources is highlighted, which can generate bias such as lack of information, as well as the lack of important data in the registration system. Another aspect that requires attention is the fact that the study did not consider the medications used by the participants during the hospitalization period. Although the study was conducted in a single hospital in the interior of the state of Rio Grande do Sul, the findings are in line with the national and international literature, which reinforces its validity. However, when interpreting these results, caution is recommended due to the particularities of the context in which the research was conducted.

This study seeks to contribute with a theoretical and practical framework relevant to the nursing area, as the results of the research demonstrated the relevance of the clinical evaluation of biomarkers by health professionals as predictors of worse prognosis, contributing to the planning of care. In addition, the findings help in clinical decision-making for people living with HIV, since they identified robust associations between the biochemical alterations presented and the occurrence of death. Actions that prioritize early diagnosis, immediate request for biochemical tests, and monitoring of these markers during all follow-up cycles of people living with HIV are recommended.

## Conclusion

It was found that people hospitalized with HIV with a VC greater than 1000 copies/mL and a low CD4+ T lymphocyte count (<200 copies/mm³) were associated with the clinical outcome of death, thus reinforcing the importance of virological and immunological control in the clinical management of HIV infection.

Another relevant finding was the association of the clinical outcome of death with metabolic and renal biomarkers, such as blood glucose levels above 126 mg/dL and high creatinine levels above 1.20 mg/dL. Finally, participants with triglyceride levels higher than 201 mg/dL represented the greatest risk factor identified in this study, reinforcing the need for strict monitoring of this parameter in this population.

Thus, according to the findings of the study, it is concluded that immunological, metabolic, renal and lipid biomarkers play a crucial role in the prognostic evaluation of people hospitalized with HIV. Thus, the early identification of these alterations is essential to assist in the implementation of more effective therapeutic strategies, aiming at reducing mortality and improving the clinical management of these people.

## Data Availability

All data generated or analysed during this study are included in this published article.
