# Supplementary material for: Associations between biomarkers and clinical outcome in people hospitalized with HIV: a retrospective cohort
Source: Rev Lat Am Enfermagem. 2026 Oct 7;34:e4952. doi: 10.1590/1518-8345.7473.4952 (PMC13679911; doi:10.1590/1518-8345.7473.4952)
Supplement: Translation

# Translation: Associações entre os biomarcadores e desfecho clínico em pessoas hospitalizadas com HIV: uma coorte retrospectiva

**DOI:** 10.1590/1518-8345.7473.4953

**Authors:** Jarbas da Silva Ziani, Jenifer Härter, Bibiana Letícia Nogara, Francielle Liz Monteiro, Laís Mara Caetano da Silva Corcini, Cláudia Zamberlan

**Affiliations:** 1 Universidade Federal de Santa Maria, Santa Maria, RS, Brasil.; 2 Universidade Federal do Pampa, Departamento de Enfermagem, Uruguaiana, RS, Brasil.; 3 Universidade Franciscana, Departamento de Biomedicina, Santa Maria, RS, Brasil.; 4 Universidade Federal de Santa Maria, Centro de Ciências da Saúde, Santa Maria, RS, Brasil.; 5 Universidade Franciscana, Departamento de Enfermagem, Santa Maria, RS, Brasil.

**Keywords:** HIV, Biomarcadores, Prognóstico, Gravidade do Paciente, Fatores de Risco, Enfermagem

## Abstract

**Objetivo:**

verificar as associações entre os biomarcadores e sua relação com o desfecho clínico das pessoas hospitalizadas com infecção pelo HIV**.**

**Método:**

coorte retrospectiva de caráter documental. Foram incluídas pessoas hospitalizadas com o diagnóstico de infecção pelo HIV, tendo como desfechos a alta hospitalar e o óbito. Avaliaram-se as variáveis sociodemográficas, clínicas e biomarcadores, por meio da análise exata de Fisher, qui-quadrado de Pearson e regressão de Cox.

**Resultados:**

entre os 102 participantes analisados, 27 (26,5%) evoluíram para óbito durante a internação. Dentre os fatores associados ao risco de óbito, destacam-se a carga viral superior a 1000 cópias/mL (HR: 3,99; p = <0,023), contagem de linfócitos T-CD4+ até 200 cópias/mm³ (HR: 1,92; p = <0,035), glicose superior a 126 mg/dL (HR: 5,40; p = <0,017), creatinina superior a 1,20 mg/dL (HR: 9,75; p = <0,012) e triglicerídeos acima de 201 mg/dL (HR: 11,75; p = <0,000).

**Conclusão:**

verificou-se que os biomarcadores imunológicos, metabólicos, renais e lipídicos desempenham um papel essencial na avaliação prognóstica de pessoas hospitalizadas com infecção pelo HIV, estando fortemente associados ao risco de óbito.

## Destaques

**(1)** Identificou-se mortalidade de 26,5% (n=27) no decorrer do estudo.

**(2)** Houve associação com o óbito entre as pessoas com carga viral acima de 1000 cópias/mL.

**(3)** Observou-se associação entre óbito e níveis de LT-CD4+ inferiores a 200 células/mL.

**(4)** Pessoas com creatinina superior a 1,20 mg/dL apresentavam 9,75 vezes maior risco.

**(5)** Triglicerídeos superiores a 201 mg/dL representaram o maior risco para óbito.

## Introdução

Globalmente, existem cerca de 39,9 milhões de pessoas vivendo com o Vírus da Imunodeficiência Humana (HIV). Destas, 1,3 milhão foram novas infecções, e 630 mil pessoas morreram devido à Síndrome da Imunodeficiência Adquirida (aids), conforme os indicadores de 2023. Ao analisar o panorama do HIV no Brasil, observa-se que, desde 2015, a epidemia está controlada. No entanto, o Programa Conjunto das Nações Unidas sobre HIV/AIDS (UNAIDS) confirmou que as taxas em adultos voltaram a aumentar desde 2000, em comparação aos anos de 1990 a 2022^1^. 

Para ampliar a qualidade e a longevidade das pessoas vivendo com HIV, foi desenvolvida a Terapia Antirretroviral (TARV), que atua na supressão da replicação viral e, consequentemente, reduz de forma significativa a morbidade e a mortalidade decorrentes da imunossupressão causada pelo HIV. Esse avanço permitiu que essas pessoas tivessem expectativa de vida semelhante à da população sem o vírus^2^. Ademais, a TARV alterou o curso da doença, transformando-a em uma condição crônica controlável, na qual as complicações não infecciosas se tornaram mais frequentes^1^ ^)-(^ ^3^.

Considerando essas mudanças e os fatores de risco para as comorbidades em pessoas vivendo com HIV, projeções globais geram preocupação. Estima-se que, até 2030, mais de 70% dessas pessoas em todo o mundo terão mais de 50 anos^4^. Isso indica que essa população está envelhecendo e, com isso, apresenta uma alta prevalência de doenças metabólicas, incluindo aumento da adiposidade, resistência à insulina, diabetes e dislipidemias, o que leva ao crescimento dos casos de Doenças Crônicas Vasculares (DCV), além de complicações renais, hepáticas, metabólicas e multimorbidades^4^ ^)-(^ ^7^.

Tendo em vista esses aspectos, a hospitalização de pessoas vivendo com HIV tornou-se um desafio global para o sistema de saúde, em especial nos países de baixa e média renda, onde o acesso aos serviços de saúde ainda é limitado. Isso, muitas vezes, leva essas pessoas a complicações graves, como infecções oportunistas e imunossupressão severa^1^ ^),(^ ^8^ ^)-(^ ^9^. A falta de diagnóstico precoce, a dificuldade de acesso à TARV e a interrupção do tratamento aumentam a vulnerabilidade dessas pessoas, resultando em internações frequentes^10^. 

Além disso, as multicomorbidades que afetam as pessoas que vivem com HIV têm elevado a demanda por serviços médicos especializados, assim como elevado os custos do tratamento e sobrecarga nos hospitais. Isso torna essencial o monitoramento dos biomarcadores nessa população, pois eles desempenham um papel fundamental na avaliação prognóstica. Biomarcadores inflamatórios, imunológicos, metabólicos, renais e lipídicos podem fornecer informações essenciais sobre a gravidade da doença, a resposta ao tratamento e a progressão clínica, ajudando a identificar precocemente aqueles com maior risco de evolução para o óbito^11^ ^)-(^ ^14^.

Diante de um levantamento realizado na literatura, identificou-se uma escassez de estudos sobre a associação entre biomarcadores e os desfechos clínicos em pessoas hospitalizadas devido ao HIV, um aspecto instigante, dado que essa temática está contemplada na agenda nacional de prioridades de pesquisa em saúde no Brasil^15^ ^)-(^ ^16^. A originalidade deste estudo reside na abordagem inovadora de explorar as variações bioquímicas em pessoas hospitalizadas, buscando estabelecer possíveis relações entre biomarcadores e prognóstico clínico. 

Além do mais, essa pesquisa se justifica pela relevância em reconhecer e identificar os fatores determinantes na evolução da aids em pessoas vivendo com HIV, contribuindo para um melhor entendimento dos mecanismos biológicos envolvidos na progressão da doença. A partir dos achados, será possível delinear estratégias eficazes para o manejo clínico, otimizar a tomada de decisão clínica e fortalecer a prática baseada em evidências. 

Dessa forma, este estudo não apenas aprimora o conhecimento científico sobre essa condição de saúde, mas também gera impactos diretos na qualidade da assistência e nos desfechos das pessoas hospitalizadas por HIV. Assim, o objetivo deste estudo é verificar as associações entre os biomarcadores e sua relação com o desfecho clínico das pessoas hospitalizadas com infecção pelo HIV.

## Método

### Delineamento e local do estudo

Trata-se de um estudo de coorte retrospectiva, de caráter documental, guiado conforme as diretrizes do *Strengthening the Reporting of Observational Studies in Epidemiology* (STROBE)^17^.

O estudo foi conduzido na rede de atenção terciária, em uma unidade de internação clínica de um hospital de média complexidade localizado em um município de grande porte do Rio Grande do Sul-RS, Brasil. A escolha do local deve-se ao fato de ser referência para o atendimento de pessoas vivendo com HIV. Além disso, o hospital possui uma profícua inserção no cenário de saúde locorregional, caracterizando-se como um hospital de ensino que presta atendimentos exclusivamente pelo Sistema Único de Saúde (SUS). 

O hospital caracteriza-se pela sua qualidade técnica e dispõe de 112 leitos de internação, distribuídos entre as unidades de internação clínica, cirúrgica, maternidade, pediatria e psiquiátrica. Nesse contexto, destaca-se que, para a pesquisa, foram analisados apenas os prontuários da enfermaria de internação clínica, que dispõe de 34 leitos, sendo 15 masculinos, 15 femininos e quatro destinados ao isolamento.

Para mais, o município do estudo está situado na região central do estado e possui uma população estimada de 271.633 habitantes em 2023. A cidade é composta por um distrito sede, que abrange oito regiões administrativas e um total de 42 bairros, além de nove distritos. No que tange à rede de saúde, a organização ocorre em três níveis gradativos de atenção, no âmbito do SUS^18^. Ainda, a investigação do cenário torna-se fundamental, considerando as elevadas taxas de HIV no município. Em 2022, estimava-se que aproximadamente 2,5 mil pessoas viviam com HIV em tal município, das quais 157 correspondiam a novos casos e cerca de 800 haviam abandonado o tratamento. Esse cenário contribui para o aumento das internações devido à progressão da aids e suas complicações clínicas^18^.

### População e período do estudo

Foram analisados todos os prontuários eletrônicos de pessoas hospitalizadas com diagnóstico de HIV no período de 01/janeiro/2022 a 31/janeiro/2023. Esse recorte temporal foi escolhido devido ao fato de que, em 2020 e 2021, 90% dos leitos da unidade estudada foram destinados ao atendimento de pessoas com COVID-19. Dessa forma, buscou-se minimizar possíveis interferências da pandemia na análise dos dados. 

### Critérios de seleção

A população do estudo foi composta por pessoas com idade superior a 18 anos, cuja hospitalização na unidade clínica tenha sido de, no mínimo, 72 horas. Quanto aos critérios de exclusão, foram desconsiderados os prontuários que apresentavam mais de 80% dos dados ilegíveis e/ou incompletos para as variáveis analisadas no estudo. 

Para identificar os prontuários elegíveis, os pesquisadores analisaram todos os prontuários disponíveis desse período, referentes às pessoas admitidas na unidade em questão, e selecionaram apenas aqueles cuja internação estava relacionada ao HIV. Ademais, para minimizar o viés de seleção, os prontuários foram analisados por dupla checagem, realizada por pesquisadores independentes.

### Instrumentos utilizados para a coleta das informações

Os dados foram coletados a partir de um instrumento elaborado especificamente para a presente pesquisa, levando-se em consideração as recomendações do protocolo clínico e diretrizes terapêuticas para manejo da infecção pelo HIV em adultos do Ministério da Saúde (MS)^19^. Além disso, o instrumento foi apreciado por cinco profissionais experientes na temática do HIV para fins de análise de conteúdo e semântica. Os especialistas receberam por e-mail o instrumento com um prazo de até 20 dias para realizar a devolutiva com as suas considerações. Os aspectos avaliados foram: ser de fácil compreensão, clareza na redação, relevância dos itens, presença de ambiguidades. Ainda, o documento continha um espaço destinado às sugestões. 

Passada essa fase, procedeu-se à realização de um teste-piloto com três prontuários elegíveis para o estudo, o qual demonstrou estar adequado para a realização da pesquisa. Ressalta-se que os prontuários do teste-piloto não foram considerados para análise do estudo. 

### Variáveis do estudo

Para a análise das variáveis bioquímicas, inicialmente foram coletados os valores preditos expressos nos exames laboratoriais. Posteriormente, esses valores foram categorizados. No que se refere às variáveis analisadas, foram coletadas características demográficas: sexo (masculino/feminino), idade em anos completos, diagnóstico de Diabetes Mellitus (DM) (sim/não), Doença Renal Crônica (DRC) (sim/não), Hipertensão Arterial Sistêmica (HAS) (sim/não), coinfecção Tuberculose/HIV (TB/HIV) (sim/não), outras comorbidades (sim/não) e desfecho (óbito/alta hospitalar). Para a análise de regressão, foram utilizados os valores brutos expressos nos exames dos participantes (sem categorização). 

Os marcadores bioquímicos hematológicos utilizados e os respectivos parâmetros foram os seguintes: carga viral (Inferior a 1000 cópias/mL/Igual ou superior a 1000 cópias/mL), linfócitos T-CD4+ (Inferior a 200 cópias/milímetro cúbico (mm^3^)/Igual ou superior a 200 cópias/mm^3^); eritrócitos (4,5 a 6,1 mm^3^/Inferior a 4,49 mm^3^), hemoglobina (inferior 12,79 miligrama (mg)/decilitro (dL)/12,8 a 17,8 mg/dL/Superior 17,9 mg/dL), Hematócrito (inferior 38,9 mg/dL/39 a 53 mg/dL/Superior 53,1 mg/dL), Leucócitos (inferior 3599 mm^3^/3600 a 11000 mm^3^/Superior 11001 mm^3^), plaquetas (inferior 149.999 mm^3^/150.000 a 450.000 mm^3^/Superior 450.001 mm^3^) e glicemia (inferior 125 mg/dL e superior a 126 mg/dL). Colesterol Total (CT) (até 190 mg/dL/Superior a 191 mg/dL), lipoproteínas responsáveis pelo transporte do colesterol. Lipoproteínas de Alta Densidade (HDL) (inferior a 39,1 mg/dL/40 a 60 mg/dL), Lipoproteína de Baixa Densidade (LDL) (100 a 159 mg/dL/Superior a 160 mg/dL), triglicerídeos (TG) (150 a 200 mg/dL/Superior a 201 mg/dL).

Já os marcadores bioquímicos de eletrólitos e função renal foram ureia (19,0 a 43,0 mg/dL/Superior a 43,1 mg/dL), creatinina (0,70 a 1,19 mg/dL/Superior a 1,20 mg/dL), sódio (137,0 a 145,9 mmol/L/Superior a 146 mmol/L), potássio (3,5 a 5,1 mmol/L/Superior a 5,2 mmol/L). Os marcadores bioquímicos de função hepática: aspartato aminotransferase (AST) (até 40,9 U/L/Superior a 41 U/L), alanina aminotransferase (ALT) (até 41 U/L/Superior a 41,1 U/L), bilirrubina total (BT) (0,3 a 1,20 mg/dL/Superior a 1,21 mg/dL), bilirrubina direta (BD) (até 0,20 mg/dL/Superior a 0,21), bilirrubina indireta (BI) (até 1,0 mg/dL/Superior a 1,1 mg/dL).

### Coleta de dados

A coleta dos dados foi realizada no período de fevereiro a junho de 2023 pelo pesquisador principal do estudo e por outra coletadora assistente, ambos devidamente capacitados. 

Para controle de vieses, tanto de aferição quanto de seleção, os prontuários foram analisados e coletados pelos dois pesquisadores e, a seguir, foram transportados para um banco de dados em planilhas no Excel^®^ (Microsoft, *version* 10, USA). Assim, os dados passaram por um processo de dupla digitação, seguido de validação para promover sua fidedignidade. Ressalta-se que não foi necessário um terceiro revisor para a análise dos prontuários.

### Tratamento e análise dos dados

Quanto à análise dos dados, utilizou-se o *software Statistical Package for the Social Sciences* (SPSS), versão 24. Aplicaram-se técnicas estatísticas descritivas e medidas de associação. As variáveis contínuas foram apresentadas pelas suas medidas de tendência central, sendo elas, média, desvio-padrão (DP) (±), mínimo e máximo. Já as variáveis categóricas foram expressas por distribuição de frequências absoluta (n) e relativa (%). Ademais, para realizar a comparação entre os grupos de acordo com o desfecho clínico, ou seja, óbito ou alta hospitalar, utilizaram-se os testes não pareados, além do teste qui-quadrado de Pearson ou o Teste Exato de Fisher, considerando a natureza das variáveis contínuas e categóricas, respectivamente.

Para determinação do risco de óbito dos participantes, utilizou-se o modelo de riscos proporcionais de Cox, o qual foi empregado para reconhecer as covariáveis que influenciaram o tempo desde o início da admissão hospitalar até o desfecho do caso. Ressalta-se que as variáveis com valor de p ≤0,05 na análise bivariada foram incluídas no modelo de análise multivariada. O ajuste global do modelo foi obtido por meio do teste da razão de verossimilhança. Verificou-se associação em relação aos marcadores bioquímicos e o desfecho (óbito e alta hospitalar) e as covariáveis associadas individualmente aos desfechos, apresentando p<0,05.

Assim, as covariáveis foram incluídas no modelo de regressão de Cox para as análises. Estabeleceu-se como um p-crítico <0,05 na análise múltipla para aceitar a associação entre o desfecho da pessoa e as alterações bioquímicas. O *Hazard Ratio* (HR) foi calculado nas análises bruta e ajustada com intervalo de confiança de 95%, e a qualidade do ajuste foi avaliada por meio da distribuição dos resíduos.

### Aspectos éticos

A pesquisa foi aprovada pelo Comitê de Ética em Pesquisa (CEP) da Universidade Franciscana (UFN) em 19/12/2022, sob o parecer n.º 5.828.312.

## Resultados

Durante o período do estudo, 1.328 pessoas foram admitidas na unidade clínica, sendo 117 internações relacionadas à infecção por HIV. Após a aplicação dos critérios de inclusão e exclusão, 15 prontuários foram desconsiderados (10 por tempo de internação inferior a 72 horas e cinco por apresentarem mais de 80,0% dos dados incompletos), resultando em 102 participantes elegíveis para análise. 

Do total da amostra, a média de idade foi de 43,3 anos (DP±12), sendo a idade mínima 18 e a máxima 78 anos. Em relação ao desfecho clínico, 27 (26,5%) casos foram a óbito no decorrer do estudo, tendo como causa o HIV, e 75 (73,5%) tiveram alta hospitalar. O tempo médio de internação registrado nos prontuários dos participantes foi de 19 dias, sendo o período mínimo de cinco e o máximo de 45 dias (DP±9).

Do total, 60 (58,8%) eram do sexo masculino e, em relação ao uso da TARV, 75 (73,5%) estavam em tratamento durante a hospitalização e 27 (16,8%) haviam interrompido. Além do mais, as características clínicas e a prevalência de comorbidades apresentadas pelos participantes estão detalhadas na Tabela 1 conforme o desfecho clínico.

**Tabela 1:** Características sociodemográficas e clínicas das pessoas hospitalizadas com HIV. Santa Maria, RS, Brasil, 2023

Variável	Desfecho clínico				
	Alta hospitalar		Óbito		
	n*	(%)^†^	n*	(%)^†^	p-valor^‡^
Sexo					**0,013** ^§^
Masculino	39	52,0%	22	81,5%	
Feminino	36	48,0%	5	18,5%	
Uso da TARV^||^					0,000^¶^
Sim	75	100%	-	-	
Não	-	-	27	100%	
Diabetes Mellitus					**0,000** ^§^
Sim	22	29,3%	17	62,9%	
Não	53	70,7%	10	37,1%	
Doença renal crônica					**0,002** ^§^
Sim	69	92,0%	18	66,7%	
Não	6	8,0%	9	33,3%	
Hipertensão arterial sistêmica					**0,000** ^§^
Sim	25	33,3%	19	70,4%	
Não	50	66,7%	8	29,6%	
Coinfecção TB/HIV**					0,357
Sim	23	30,7%	11	40,7%	
Não	52	69,3	16	59,3%	
Outras comorbidades					**0,028** ^¶^
Sim	25	33,3%	4	14,8%	
Não	50	66,7%	23	85,2%	

*n = Tamanho da amostra conforme o desfecho; ^†^% = Porcentagem; ^‡^p-valor = Valor estatisticamente significante no nível de 5% (p ≤ 0,05); ^§^Teste qui-quadrado de Pearson; ^||^TARV = Terapia Antirretroviral; ^¶^Teste de Exato de Fisher; **TB/HIV = Tuberculose/Vírus da Imunodeficiência Humana

Em relação ao motivo da internação dos participantes na unidade de internação clínica, identificou-se: imunossupressão - 30 pessoas (29,4%), pneumonia - 10 (9,8%), tuberculose - seis (5,9%), infecção oportunista em investigação - quatro (3,9%), criptococose - três (2,9%), desnutrição - três (2,9%), hepatites virais - três (2,9%), herpes-zoster - três (2,9%), pancreatite - três (2,9%), sarcoma de Kaposi - três (2,9%), abuso sexual - dois (2,0%), candidíase oral e esofágica - dois (2,0%), neurotoxoplasmose - dois (2,0%), pneumocistose - dois (2,0%), sepse - dois (2,0%) e outros - 24 (23,5%).

A Tabela 2 apresenta e compara as diferenças entre os marcadores hematológicos dos participantes do estudo e a sua relação com os desfechos clínicos. Observou-se uma associação estatística entre o óbito e os participantes que apresentaram contagem de eritrócitos inferiores a 4,49/mm^3^, níveis de hemoglobina abaixo de 12,79 mg/dL e plaquetas inferiores a 149.999 mm^3^, com valor de p=0,000. 

**Tabela 2:** Associação dos biomarcadores hematológicos e do perfil lipídico em pessoas hospitalizadas com HIV, segundo o desfecho clínico. Santa Maria, RS, Brasil, 2023

Variável	Desfecho clínico				
	Alta hospitalar		Óbito		
	n*	(%)^†^	n*	(%)^†^	p-valor^‡^
Eritrócitos (mm^3§^)					**0,000** ^||^
4,5 a 6,1	51	50,0%	3	2,9%	
Inferior a 4,49	24	23,5%	24	23,6%	
Hemoglobina (mg/dL^¶^)					**0,000** ^||^
Inferior a 12,79	21	20,7%	25	24,5%	
12,8 a 17,8	54	52,9%	2	2,0%	
Hematócrito (mg/dL^¶^)					0,321**
Inferior a 38,9	67	65,7%	22	21,6%	
39 a 53	8	7,8%	5	4,9%	
Leucócitos (mm^3§^)					0,836**
Inferior a 3599	11	10,8%	4	3,9%	
3600 a 11000	46	45,2%	15	14,7%	
Superior a 11001	18	17,6%	8	7,8%	
Plaquetas (mm^3§^)					**0,000** ^||^
Inferior a 149.999	11	10,8%	14	13,7%	
150.000 a 450.000	59	57,8%	10	9,8%	
Superior a 450.001	5	4,9%	3	2,9%	
Colesterol Total (mg/dL^¶^)					**0,000** ^||^
até 190	53	52,0%	3	2,9%	
Superior a 191	22	21,6%	24	23,5%	
HDL^††^ (mg/dL^¶^)					**0,000** ^||^
Inferior a 39,9	38	37,2%	26	25,5%	
40 a 60	37	36,3%	1	1,0%	
LDL^‡‡^ (mg/dL^¶^)					**0,000** ^||^
100 a 159	47	46,1%	3	2,9%	
Superior a 160	28	27,5%	24	23,5%	
Triglicerídeos (mg/dL^¶^)					**0,000** ^||^
150 a 200	64	61,7%	1	1,0%	
Superior a 201	11	10,8%	27	26,5%	

*n = Tamanho da amostra conforme o desfecho; ^†^% = Porcentagem; ^‡^p-valor = Valor estatisticamente significante no nível de 5% (p ≤ 0,05); ^§^mm^3^ = Milímetros cúbicos; ^||^Teste de Exato de Fisher; ^¶^mg/dL = Miligramas por decilitros; **Teste qui-quadrado de Pearson; ^††^HDL = Lipoproteínas de alta densidade; ^‡‡^LDL = Lipoproteína de baixa densidade

Além disso, verificou-se uma associação entre o perfil lipídico e o desfecho clínico. Participantes com colesterol total acima de 191 mg/dL, HDL inferior a 39,9 mg/dL, LDL superior a 160 mg/dL e triglicerídeos acima de 201 mg/dL apresentaram maior associação com óbito (p=0,000).

Quanto aos marcadores da função renal dos participantes, identificou-se que há associação estatística do desfecho óbito com os marcadores de ureia, creatinina, sódio e potássio, conforme destacado na Tabela 3.

**Tabela 3:** Associação dos biomarcadores renais em pessoas hospitalizadas com HIV, segundo o desfecho clínico. Santa Maria, RS, Brasil, 2023

Variável	Desfecho clínico				
	Alta hospitalar		Óbito		
	n*	(%)^†^	n*	(%)^†^	p-valor^‡^
Ureia (mg/dL^§^)					**0,000** ^||^
9,0 a 43,0	8	7,8%	20	19,6%	
Superior a 43,1	67	65,7%	7	6,9%	
Creatinina (mg/dL^§^)					**0,000** ^¶^
0,70 a 1,19	62	60,8%	4	3,9%	
Superior a 1,20	13	12,7%	23	22,6%	
Sódio (mmol/L**)					**0,000** ^||^
137,0 a 145,9	69	67,6%	10	9,8%	
Superior a 146	6	5,9%	17	16,7%	
Potássio (mmol/L^**^)					**0,000** ^¶^
3,5 a 5,1	71	69,6%	6	5,9%	
Superior a 5,2	4	3,9%	21	20,6%	

*n = Tamanho da amostra conforme o desfecho; ^†^% = Porcentagem; ^‡^p-valor= Valor estatisticamente significante no nível de 5% (p ≤ 0,05); ^§^mg/dL = Miligramas por decilitros; ^||^Teste qui-quadrado de Pearson; ^¶^Teste de Exato de Fisher; **mmol/L = Milimol por litro

Conforme a Tabela 4, a evolução para o óbito demonstrou associação significativa com os marcadores hepáticos. O grupo de óbito apresentou maior prevalência de resultados que indicam pior prognóstico, especificamente para AST (p=0,000), ALT (p=0,001), BT (p=0,003) e BD (p=0,032).

**Tabela 4:** Associação dos biomarcadores hepáticos em pessoas hospitalizadas com HIV, segundo o desfecho clínico. Santa Maria, RS, Brasil, 2023

Variável	Desfecho clínico				
	Alta hospitalar		Óbito		
	n*	(%)^†^	N*	(%)^†^	p-valor^‡^
AST^§^ (U/L^||^)					**0,000** ^¶^
até 40,9	47	46,1%	4	3,9%	
Superior a 41	28	27,5%	23	22,5%	
ALT** (U/L^||^)					**0,001** ^††^
até 41	60	58,8%	10	9,8%	
Superior 41,1	15	14,7%	17	16,7%	
Bilirrubina total (mg/dL^‡‡^)					**0,003** ^††^
0,3 a 1,20	41	40,2%	5	4,9%	
Superior 1,21	34	33,3%	22	21,6%	
Bilirrubina direta (mg/dL^‡‡^)					0,052^††^
até 0,20	45	44,1%	12	11,8%	
Superior 0,21	30	29,4%	15	14,7%	
Bilirrubina indireta (mg/dL^‡‡^)					**0,032** ^¶^
até 1,0	22	21,6%	2	2,0%	
Superior a 1,1	53	52,0%	25	24,5%	

*n = Tamanho da amostra conforme o desfecho; ^†^% = Porcentagem; ^‡^p-valor = Valor estatisticamente significante no nível de 5% (p ≤ 0,05); ^§^AST = Aspartato aminotransferase; ^||^U/L = Unidades por litro; ^¶^Teste de Exato de Fisher; **ALT = Alanina aminotransferase; ^††^Teste qui-quadrado de Pearson; ^‡‡^mg/dL = Miligramas por decilitros

Conforme dados expressos na Tabela 5, o modelo ajustado identificou associação com o óbito entre as pessoas que possuíam CV igual ou superior a 1000 cópias/mL (p=0,023), linfócitos T-CD4 igual ou inferior a 200 células/mm^3^ (p=0,035), glicemia igual ou superior a 126 mg/dL (p=0,017), creatinina igual ou superior a 1,20 mg/dL (p=0,012) e triglicerídeos igual ou superior a 201 mg/dL (p=0,000).

**Tabela 5:** Análise de regressão de Cox bruta e ajustada para o risco de óbito em pessoas hospitalizadas com HIV. Santa Maria, RS, Brasil, 2023

Variável	HR* bruta (IC 95%^†^)	p^‡^	HR* ajustada (IC 95%^†^)	p^‡^
Carga viral (cópias/mL)				
Igual ou superior a 1000	9,78 (3,23-12,43)	0,000	3,99 (1,25-12,85)	**0,023** ^§^
L T-CD4+^||^ (cópias/mm^3¶^)				
igual ou inferior a 200 células/mm^3^	4,12 (1,78-2,12)	0,008	1,92 (1,85-2,36)	**0,035** ^§^
Glicose (mg/dL**)				
Superior a 126	4,49 (2,29-8,36)	0,005	5,40 (2,11-8,21)	**0,017** ^§^
Ureia (mg/dL**)				
Superior a 43,1	0,16 (0,06-1,26)	0,007	0,19 (0,09-1,99)	0,195
Creatinina (mg/dL**)				
Superior a 1,20	10,12 (5,23-15,36)	0,009	9,75 (2,56-14,98)	**0,012** ^§^
Sódio (mmol/L^††^)				
Superior a 146	2,19 (1,34-3,60)	0,009	2,12 (1,27-3,51)	0,051
Potássio (mmol/L^††^)				
Superior a 5,2	3,25 (0,81-18,95)	0,007	5,23 (0,85-45,69)	0,089
Colesterol Total (mg/dL**)				
Superior a 191	0,52 (0,08-2,36)	0,001	0,23 (0,23-2,15)	0,212
HDL^‡‡^ (mg/dL**)				
Inferior a 39,9	2,45 (0,88-6,33)	0,008	0,68 (0,23-1,65)	0,654
LDL^§§^ (mg/dL**)				
Superior a 160	2,45 (0,85-5,71)	0,004	1,63 (0,63-4,71)	0,854
Triglicerídeos (mg/dL**)				
Superior a 201	12,15 (2,75-15,63)	0,008	11,75 (4,18-23,26)	**0,000** ^§^

*HR = *Hazard Ratio*; ^†^IC = Intervalo de confiança de 95%; ^‡^p = Nível de significância; ^§^Resultado estatisticamente significativo; ^||^L T-CD4+ = Linfócitos T-CD4+; ^¶^mm^3^ = Milímetros cúbicos; **mg/dL = Miligramas por decilitros; ^††^mmol/L = Milimol por litro; ^‡‡^HDL = Lipoproteínas de alta densidade; ^§§^LDL = Lipoproteína de baixa densidade

Além disso, observa-se que os participantes com resultados de CV acima de 1000 cópias/mL apresentavam, aproximadamente, 3,99 vezes mais risco de evoluir para óbito, em comparação com aqueles que possuíam resultados inferiores a 1000 cópias/mL (HR ajustado: 3,99; IC 95%: 1,25-12,85). Pessoas que tiveram uma contagem de linfócitos T-CD4+ inferior a 200 células/mm^3^ tiveram 1,92 mais risco de vir a óbito em comparação com quem possuía contagem igual ou superior a 200 mm^3^ (HR ajustado: 1,92; IC 95%: 1,85-2,36).

Participantes que apresentaram resultado superior a 126 mg/dL no biomarcador da glicemia tiveram 5,40 vezes mais risco de vir a óbito durante a hospitalização, quando comparado com pessoas que apresentaram valor inferior a 125 mg/dL (HR ajustada: 5,40; IC 95%: 2,11-8,21). Observou-se que participantes com valor superior a 1,20 mg/dL no marcador de creatinina possuíam 9,75 vezes mais risco de óbito, em comparação aos que possuíam valores inferiores a 1,19 mg/dL (HR ajustada: 9,75; IC 95%: 2,56-14-98). 

Ainda, os participantes que tiveram taxas de triglicerídeos acima de 201 mg/dL possuíram um risco de óbito 11,75 vezes maior que aqueles que exibiram valores menores (HR ajustado: 11,75; IC 95%: 4,18-23,26).

## Discussão

Objetivando analisar as alterações entre os biomarcadores apresentados pelas pessoas hospitalizadas com infecção pelo HIV, observa-se que os valores de CV reforçam o que já vem sendo identificado na literatura científica. Estudos indicam que as pessoas vivendo com HIV em uso da TARV, com carga viral indetectável ou entre 200 e 1000 cópias/mL, apresentam melhores prognósticos da infecção^20^. Em contrapartida, pessoas hospitalizadas com CV superior a 1000 cópias/mL estão diretamente associadas a marcadores avançados de aids e possuem maior risco de evolução para óbito^21^. 

Nesse contexto, a presente pesquisa reforça que pessoas vivendo com HIV que apresentam contagens de linfócitos T-CD4+ inferiores a 200 cópias/mm³ estão mais suscetíveis a infecções oportunistas e, consequentemente, possuem maior probabilidade de evolução para óbito devido à doença. Um estudo brasileiro que analisou o perfil de óbito de 109.636 pessoas vivendo com HIV no período de 2014 a 2019 identificou resultados similares aos achados do presente estudo, mostrando que menores taxas de linfócitos T-CD4+ estavam associadas à mortalidade^22^. Ainda, resultados desta pesquisa demonstram que pessoas com contagem de linfócitos T-CD4+ inferiores a 200 cópias/mm^3^ possuem, aproximadamente, 1,92 vezes mais risco de evoluir para o óbito em comparação àqueles com valores igual ou superior a 200 mm³ (p=0,035).

Da mesma forma, estudo realizado na Tanzânia identificou que as pessoas que vivem com HIV possuem um risco até cinco vezes maior de desenvolver DM em comparação àqueles que não vivem com o vírus^23^. Essa associação se torna ainda mais expressiva ao considerar os achados da presente pesquisa, na qual os participantes com níveis glicêmicos superiores a 126 mg/dL apresentaram um risco de 5,4 vezes mais chances de vir a óbito. Ressalta-se que o aumento nos níveis glicêmicos em pessoas vivendo com HIV pode ser explicado, em grande parte, pela resistência insulínica induzida pela TARV, fator que contribui para o desenvolvimento da DM^23^. 

Além disso, destaca-se que, logo após a introdução da TARV, as pessoas vivendo com HIV experimentam a reconstituição imunológica, um processo que pode resultar no aumento da secreção dos fatores de necrose tumoral alfa (TNF-α). Esse fenômeno pode induzir resistência à insulina por meio de diversos mecanismos que envolvem a cascata de sinalização do receptor de insulina^24^. Diante disso, torna-se essencial que os profissionais de saúde mantenham um compromisso contínuo com as estratégias preventivas, incluindo a monitorização regular dos níveis glicêmicos. Essa abordagem visa reduzir a progressão para o DM, prevenindo o duplo fardo da doença nessa população e, consequentemente, diminuindo as chances de um pior prognóstico^23^.

A análise da associação entre os marcadores renais e o desfecho de óbito revelou uma associação significativa entre os marcadores renais e o risco de óbito. Pessoas com valores superiores a 1,20 mg/dL apresentaram um risco de 9,75 vezes maior de evolução para o óbito. Diante disso, é fundamental ressaltar que o comprometimento renal representa um problema grave em pessoas vivendo com HIV, estando associado a diversas causas, incluindo nefropatias decorrentes do HIV, nefrotoxicidade por medicamentos, procedimentos médicos e, principalmente, à doença renal crônica, que pode ser causada ou agravada por comorbidades^25^ ^)-(^ ^26^. 

Um estudo realizado nos Estados Unidos apontou que pessoas vivendo com HIV apresentam um risco de até 13 vezes maior de desenvolver insuficiência renal em comparação com pessoas que não vivem com o vírus, um valor superior ao identificado no presente estudo^27^. Essa divergência pode estar relacionada ao acesso gratuito e universal ao tratamento no Brasil, o que pode contribuir para um melhor controle das complicações renais nessa população.

Além disso, o estudo sugere que todas as pessoas diagnosticadas com HIV devem ser submetidas a uma avaliação detalhada acerca das alterações renais, considerando a possibilidade de coexistência de múltiplos fatores desencadeantes^27^. Essa recomendação está em consonância com os protocolos nacionais, que visam garantir um monitoramento adequado das complicações associadas ao HIV^28^. 

Os marcadores do perfil lipídico demonstraram ser relevantes no estudo, pois estavam associados ao desfecho de óbito. Nos Estados Unidos, um estudo conduzido com 981 pessoas vivendo com HIV identificou resultados semelhantes, mostrando que 41% dos participantes tinham níveis elevados de CT (p=0,000) e TG, enquanto 21,4% das pessoas que evoluíram para o óbito possuíam associação com esses marcadores (p=0,002)^4^. Dessa forma, nota-se que o perfil lipídico encontrado nesta pesquisa apresenta valores superiores aos do estudo mencionado, porém, mantém a mesma associação significativa com o óbito como desfecho (p=0,000). 

Estudo brasileiro^22^ alerta sobre a importância de os profissionais de saúde considerarem estratégias voltadas para o autocuidado e a adoção de hábitos de vida saudáveis, visando minimizar esses fatores de risco. Tais fatores estão diretamente relacionados a complicações cardiovasculares, que representam a principal causa de morbidade e mortalidade entre as pessoas vivendo com HIV^29^. 

Adicionalmente, a alta prevalência de comorbidades na amostra do presente estudo, com 38,2% de diagnósticos de DM, 41,2% de HAS e 85,3% de DRC, está em consonância com o que tem sido observado globalmente nas pessoas que vivem com HIV. A evolução na prescrição de medicamentos para doenças crônicas e a crescente prevalência dessas comorbidades entre essas pessoas, conforme demonstrado em um estudo recente na Bélgica, sublinham a necessidade de monitoramento contínuo e gestão adequada para evitar desfechos desfavoráveis^30^. A presença dessas comorbidades, por si só, já é um fator de pior prognóstico, e sua interação com as alterações bioquímicas discutidas neste estudo pode intensificar o risco de óbito.

Diante dos achados desta pesquisa, corroborados pela literatura científica^4^ ^),(^ ^29^, destaca-se a importância da adesão à TARV e a necessidade do desenvolvimento de hábitos de vida saudáveis para minimizar desfechos clínicos desfavoráveis. Embora a TARV esteja associada a um maior risco de alterações metabólicas e ao aumento da probabilidade de desenvolvimento de doenças cardiovasculares a longo prazo, ela continua sendo a estratégia mais eficaz para o manejo da infecção pelo HIV, proporcionando melhores prognósticos e qualidade de vida às pessoas que vivem com HIV^31^. 

A prevalência de doença hepática em pessoas vivendo com HIV varia entre 4% e 18% em diferentes regiões do mundo, sendo que as taxas de mortalidade relacionadas a danos hepáticos são até 10 vezes mais frequentes nessa população em comparação à população geral^32^. Estudo realizado na Espanha reafirmou esse achado, confirmando que os participantes que viviam com HIV e possuíam comprometimento hepático tinham pior prognóstico em relação àqueles sem a infecção pelo vírus. As principais causas de doença hepática nessa população incluíram coinfecções pelo vírus da Hepatite C (HCV) e o vírus da Hepatite B (HBV), lesão hepática induzida por medicamentos, doença alcoólica e doença hepática gordurosa não alcoólica^33^. 

Ainda os autores ressaltam a importância do comprometimento dos profissionais de saúde, desde a fase inicial do diagnóstico da infecção, com o monitoramento dos marcadores hepáticos e lipídicos, independente da combinação da TARV utilizada^33^. Esse acompanhamento é essencial para reduzir o risco de progressão da doença hepática e o desenvolvimento de carcinoma hepatocelular.

Diante dos aspectos destacados, aponta-se a relevância da prática clínica e da realização de pesquisas futuras acerca do tema, pois a interrupção do tratamento, recidiva e a coinfecção por HCV em pessoas vivendo com HIV exigem um acompanhamento rigoroso, especialmente no que se refere à fibrose residual. Além disso, estudos bem delineados que investiguem o desenvolvimento de distúrbios metabólicos a longo prazo em pessoas coinfectadas podem contribuir significativamente para a redução da magnitude desse problema, auxiliando na elaboração de estratégias terapêuticas mais eficazes^33^.

A análise dos motivos de internação dos participantes, que incluiu infecções oportunistas como pneumonia (9,9%), tuberculose (5,9%), criptococose (2,9%) e sepse (2,0%), reforça a importância da atenção à imunossupressão severa. A diversidade desses quadros clínicos sublinha a vulnerabilidade dessa população a complicações graves, o que pode explicar a forte associação entre os biomarcadores alterados e o desfecho de óbito^8^ ^)-(^ ^9^.

Além da análise dos marcadores que se mantiveram no modelo de regressão, este estudo também investigou a relevância dos biomarcadores hematológicos. Embora não tenham apresentado poder estatístico para se manterem no modelo, esses biomarcadores são de grande importância na imunidade de pessoas que vivem com HIV. A análise dos resultados dos exames de hemograma dos participantes revelou uma associação significativa entre a presença de anemia e a evolução para óbito p=0,000. Este achado reforça os resultados de uma meta-análise, que identificou a presença de anemia em 78,8% das pessoas vivendo com HIV, associando essa condição a piores prognósticos de saúde^34^. 

Desse modo, destaca-se a importância da realização de exames periódicos em pessoas vivendo com HIV, bem como da atenção dos profissionais de saúde às suas alterações, uma vez que a anemia vem sendo citada como um marcador de piores prognósticos da infecção e está associada principalmente à redução da sobrevida dessas pessoas^34^. É pertinente ressaltar que diversos fatores podem influenciar o desenvolvimento da anemia em pessoas vivendo com HIV, incluindo o uso da TARV, a presença de mediadores inflamatórios e as coinfecções associadas à infecção. Esses fatores podem comprometer a proliferação e diferenciação das células-tronco/progenitoras hematopoiéticas durante a hematopoiese, culminando no quadro clínico de anemia^35^.

No que se refere às limitações do estudo, destaca-se a utilização de fonte secundária, o que pode gerar viés como a falta de informações, bem como a carência de dados importantes no sistema de registro. Outro aspecto que requer atenção é o fato de o estudo não ter considerado as medicações em uso pelos participantes durante o período de internação. Embora o estudo tenha sido conduzido em um único hospital no interior do estado do RS, os achados estão em consonância com a literatura nacional e internacional, o que reforça sua validade. No entanto, ao interpretar esses resultados, recomenda-se cautela em função das particularidades do contexto em que a pesquisa foi realizada. 

Este estudo busca contribuir com um arcabouço teórico e prático relevante para a área da enfermagem, pois os resultados da pesquisa demonstraram a relevância da avaliação clínica dos biomarcadores pelos profissionais de saúde como marcadores preditores de piores prognósticos, contribuindo para o planejamento da assistência. Ainda, os achados auxiliam na tomada de decisões clínicas para as pessoas que vivem com HIV, uma vez que identificaram associações robustas entre as alterações bioquímicas apresentadas e a ocorrência do óbito. Recomenda-se ações que priorizem o diagnóstico precoce, a solicitação imediata dos exames bioquímicos e o monitoramento destes marcadores durante todos os ciclos de acompanhamento das pessoas vivendo com HIV.

## Conclusão

Verificou-se que pessoas hospitalizadas com HIV com CV superior a 1000 cópias/mL e a baixa contagem de linfócitos T-CD4+ (<200 cópias/mm³) estavam associadas ao desfecho clínico de óbito, reforçando, assim, a importância do controle virológico e imunológico no manejo clínico da infecção pelo HIV. 

Outro achado relevante foi a associação do desfecho clínico de óbito aos biomarcadores metabólicos e renais, como níveis de glicemia superiores a 126 mg/dL e níveis elevados de creatinina superiores a 1,20 mg/dL. Por fim, os participantes que apresentaram níveis de triglicerídeos superiores a 201 mg/dL representaram o maior fator de risco identificado neste estudo, reforçando a necessidade de um acompanhamento rigoroso desse parâmetro nessa população.

Assim, conforme os achados do estudo, conclui-se que os biomarcadores imunológicos, metabólicos, renais e lipídicos desempenham um papel crucial na avaliação prognóstica de pessoas hospitalizadas com HIV. Desse modo, torna-se fundamental a identificação precoce dessas alterações para auxiliar na implementação de estratégias terapêuticas mais eficazes, visando a redução da mortalidade e a melhoria do manejo clínico dessas pessoas.

## Data Availability

Todos os dados gerados ou analisados durante este estudo estão incluídos neste artigo publicado.

# Translation: Asociaciones entre biomarcadores y resultados clínicos en personas hospitalizadas con VIH: una cohorte retrospectiva

**DOI:** 10.1590/1518-8345.7473.4951

**Authors:** Jarbas da Silva Ziani, Jenifer Härter, Bibiana Letícia Nogara, Francielle Liz Monteiro, Laís Mara Caetano da Silva Corcini, Cláudia Zamberlan

**Affiliations:** 1 Universidade Federal de Santa Maria, Santa Maria, RS, Brasil.; 2 Universidade Federal do Pampa, Departamento de Enfermagem, Uruguaiana, RS, Brasil.; 3 Universidade Franciscana, Departamento de Biomedicina, Santa Maria, RS, Brasil.; 4 Universidade Federal de Santa Maria, Centro de Ciências da Saúde, Santa Maria, RS, Brasil.; 5 Universidade Franciscana, Departamento de Enfermagem, Santa Maria, RS, Brasil.

**Keywords:** VIH, Biomarcadores, Pronóstico, Gravedad del Paciente, Factores de Riesgo, Enfermería

## Abstract

**Objetivo:**

verificar las asociaciones entre los biomarcadores y su relación con el resultado clínico de las personas hospitalizadas con infección por VIH.

**Método:**

cohorte retrospectiva de personajes documentales. Se incluyeron las personas hospitalizadas con diagnóstico de infección por VIH, con el alta hospitalaria y la muerte como consecuencias. Se evaluaron variables sociodemográficas, clínicas y biomarcadoras utilizando el análisis exacto de Fisher, el qui-cuadrado de Pearson y la regresión de Cox.

**Resultados:**

entre los 102 participantes analizados, 27 (26,5%) fallecieron durante la hospitalización. Entre los factores asociados al riesgo de muerte, destacan la carga viral superior a 1000 copias/mL (HR: 3,99; p = <0,023), recuento de linfocitos T CD4+ hasta 200 copias/mm³ (HR: 1,92; p = <0,035), glucosa superior a 126 mg/dL (HR: 5,40; p = <0,017), creatinina superior a 1,20 mg/dL (HR: 9,75; p = <0,012) y triglicéridos superiores a 201 mg/dL (HR: 11,75; p = <0,000).

**Conclusión:**

se encontró que los biomarcadores inmunológicos, metabólicos, renales y lipídicos desempeñan un papel esencial en la evaluación pronóstica de personas hospitalizadas con infección por VIH, estando fuertemente asociados con el riesgo de muerte.

## Destacados

**(1)** Se identificó una tasa de mortalidad del 26,5% (n=27) durante el estudio.

**(2)** Había una asociación con la muerte entre personas con una carga viral superior a 1000 copias/mL.

**(3)** Se observó una asociación entre la muerte y los niveles de LT-CD4+ inferiores a 200 células/mL.

**(4)** Las personas con creatinina superior a 1,20 mg/dL tenían un riesgo 9,75 veces mayor.

**(5)** Los triglicéridos superiores a 201 mg/dL representaron el mayor riesgo de muerte.

## Introducción

A nivel mundial, hay alrededor de 39,9 millones de personas que viven con el Virus de la Inmunodeficiencia Humana (VIH). De estos, 1,3 millones fueron nuevas infecciones y 630 mil personas murieron debido al Síndrome de Inmunodeficiencia Adquirida (SIDA), según los indicadores de 2023. Al analizar el panorama del VIH en Brasil, se observa que, desde 2015, la epidemia está bajo control. Sin embargo, el Programa Conjunto de las Naciones Unidas sobre VIH/SIDA (ONUSIDA) ha confirmado que las tasas en adultos han vuelto a aumentar desde el año 2000, en comparación con los años 1990 a 2022^1^.

Para aumentar la calidad y longevidad de las personas que viven con VIH, se desarrolló la Terapia Antirretroviral (TAR), que actúa suprimiendo la replicación viral y, en consecuencia, reduce significativamente la morbilidad y mortalidad resultantes de la inmunosupresión causada por el VIH. Este avance permitió a estas personas tener una esperanza de vida similar a la de la población sin el virus^2^. Además, la TAR alteró el curso de la enfermedad, transformándola en una condición crónica controlable, en la que las complicaciones no infecciosas se hicieron más frecuentes^1^ ^)-(^ ^3^.

Teniendo en cuenta estos cambios y los factores de riesgo de comorbilidades en personas que viven con VIH, las proyecciones globales son preocupantes. Se estima que, para 2030, más del 70% de estas personas en el mundo tendrán más de 50 años^4^. Esto indica que esta población está envejeciendo y, por tanto, presenta una alta prevalencia de enfermedades metabólicas, incluyendo aumento de la adiposidad, resistencia a la insulina, diabetes y dislipidemias, lo que conduce a un aumento de casos de Enfermedades Vasculares Crónicas (ECV), además de complicaciones renales, hepáticas, metabólicas y multimorbilidades^4^ ^)-(^ ^7^.

En vista de estos aspectos, la hospitalización de personas que viven con VIH se ha convertido en un desafío global para el sistema de salud, especialmente en países de ingresos bajos y medios, donde el acceso a los servicios de salud sigue siendo limitado. Esto suele llevar a estas personas a complicaciones graves, como infecciones oportunistas y una inmunosupresión severa^1^ ^),(^ ^8^ ^)-(^ ^9^. La falta de un diagnóstico precoz, la dificultad para acceder a la TAR y la interrupción del tratamiento aumentan la vulnerabilidad de estas personas, lo que resulta en hospitalizaciones frecuentes^10^.

Además, las multicomorbilidades que afectan a las personas con VIH han incrementado la demanda de servicios médicos especializados, así como los costes de tratamiento y la sobrecarga hospitalaria. Esto hace esencial monitorizar los biomarcadores en esta población, ya que desempeñan un papel clave en la evaluación pronóstica. Los biomarcadores inflamatorios, inmunológicos, metabólicos, renales y lipídicos pueden proporcionar información esencial sobre la gravedad de la enfermedad, la respuesta al tratamiento y la progresión clínica, ayudando a identificar temprano a quienes tienen mayor riesgo de muerte^11^ ^)-(^ ^14^.

A la luz de una encuesta realizada en la literatura, se identificó la falta de estudios sobre la asociación entre biomarcadores y resultados clínicos en personas hospitalizadas por VIH, un aspecto impulsor, dado que este tema está incluido en la agenda nacional de prioridades de investigación sanitaria en Brasil^15^ ^)-(^ ^16^. La originalidad de este estudio radica en el enfoque innovador de explorar las variaciones bioquímicas en personas hospitalizadas, buscando establecer posibles relaciones entre biomarcadores y pronóstico clínico.

Además, esta investigación se justifica por la relevancia de reconocer e identificar los factores determinantes en la evolución del SIDA en personas que viven con VIH, contribuyendo a una mejor comprensión de los mecanismos biológicos implicados en la progresión de la enfermedad. A partir de los hallazgos, será posible delinear estrategias eficaces para la gestión clínica, optimizar la toma de decisiones clínicas y fortalecer la práctica basada en la evidencia. 

Así, este estudio no solo mejora el conocimiento científico sobre esta condición de salud, sino que también genera impactos directos en la calidad de la atención y los resultados de las personas hospitalizadas por VIH. Por tanto, el objetivo de este estudio es verificar las asociaciones entre los biomarcadores y su relación con el resultado clínico de las personas hospitalizadas con infección por VIH.

## Método

### Diseño y lugar del estudio

Se trata de un estudio de cohorte retrospectivo y documental, guiado por las directrices del programa *Strengthening the Reporting of Observational Studies in Epidemiology* (STROBE)^17^.

El estudio se realizó en la red de atención terciaria, en una unidad clínica de hospitalización de un hospital de complejidad media situado en un gran municipio de Rio Grande do Sul-RS, Brasil. La elección de la ubicación se debe a que es una referencia para el cuidado de personas que viven con VIH. Además, el hospital cuenta con una inserción fructífera en el panorama sanitario local y regional, caracterizándose como un hospital docente que ofrece atención exclusivamente a través del Sistema Único de Salud (SUS).

El hospital se caracteriza por su calidad técnica y cuenta con 112 camas de hospitalización, distribuidas entre las unidades clínicas, quirúrgicas, de maternidad, pediátrica y psiquiátrica. En este contexto, cabe destacar que, para la investigación, solo se analizaron los historiales médicos de la planta de hospitalización clínica, que cuenta con 34 camas, 15 masculinas, 15 femeninas y cuatro para aislamiento.

Además, el municipio del estudio se encuentra en la región central del estado y tiene una población estimada de 271.633 habitantes en 2023. La ciudad está compuesta por una sede de distrito, que abarca ocho regiones administrativas y un total de 42 barrios, además de nueve distritos. En cuanto a la red sanitaria, la organización se desarrolla en tres niveles graduales de atención, dentro del alcance del SUS^18^. Además, la investigación del escenario se vuelve fundamental, considerando las altas tasas de VIH en el municipio. En 2022, se estimó que aproximadamente 2.500 personas vivían con VIH en este municipio, de las cuales 157 correspondían a nuevos casos y alrededor de 800 habían abandonado el tratamiento. Este escenario contribuye al aumento de hospitalizaciones debido a la progresión del SIDA y sus complicaciones clínicas^18^.

### Población y periodo de estudio

Se analizaron todos los historiales médicos electrónicos de las personas hospitalizadas con diagnóstico de VIH desde el 1 de enero de 2022 hasta el 31 de enero de 2023. Este periodo se eligió debido a que, en 2020 y 2021, el 90% de las camas de la unidad estudiada estaban destinadas al cuidado de personas con COVID-19. De este modo, buscamos minimizar la posible interferencia de la pandemia en el análisis de datos. 

### Criterios de selección

La población del estudio estaba compuesta por personas mayores de 18 años, cuyo ingreso en la unidad clínica fue al menos 72 horas. En cuanto a los criterios de exclusión, los historiales médicos que presentaron más del 80% de los datos eran ilegibles y/o incompletos para las variables analizadas en el estudio. 

Para identificar los historiales médicos elegibles, los investigadores analizaron todos los registros médicos disponibles de ese periodo, refiriéndose a las personas ingresadas en la unidad en cuestión, y seleccionaron solo a aquellas cuya hospitalización estaba relacionada con el VIH. Además, para minimizar el sesgo de selección, los historiales médicos se analizaron mediante doble verificación, realizada por investigadores independientes.

### Instrumentos utilizados para la recopilación de información

Se recogieron datos de un instrumento desarrollado específicamente para el presente estudio, teniendo en cuenta las recomendaciones del protocolo clínico y las directrices terapéuticas para el manejo de la infección por VIH en adultos del Ministerio de la Salud (MS)^19^. Además, el instrumento fue apreciado por cinco profesionales con experiencia en el tema del VIH para fines de contenido y análisis semántico. Los especialistas recibieron el instrumento por correo electrónico con un plazo de hasta 20 días para devolver sus consideraciones. Los aspectos evaluados fueron: facilidad de entendimiento, claridad en la escritura, relevancia de los elementos y presencia de ambigüedades. Además, el documento contenía un espacio para sugerencias.

Tras esta fase, se realizó una prueba piloto con tres historiales médicos aptos para el estudio, que resultaron adecuados para la investigación. Cabe destacar que los registros médicos de la prueba piloto no se consideraron para el análisis del estudio.

### Variables del estudio

Para el análisis de las variables bioquímicas, se recogieron inicialmente los valores predichos expresados en las pruebas de laboratorio. Posteriormente, estos valores fueron categorizados. Se recopilaron características demográficas en función de las variables analizadas: género (hombre/mujer), edad en años completos, diagnóstico de diabetes mellitus (DM) (sí/no), enfermedad renal crónica (ERC) (sí/no), hipertensión arterial sistémica (SAH) (sí/no), coinfección tuberculosis/VIH (TB/VIH) (sí/no), otras comorbilidades (sí/no) y resultado (muerte/alta hospitalaria). Para el análisis de regresión, se utilizaron los valores brutos expresados en los exámenes de los participantes (sin categorización).

Los marcadores bioquímicos hematológicos utilizados y sus respectivos parámetros fueron los siguientes: carga viral (menos de 1000 copias/mL/igual o superior a 1000 copias/mL), linfocitos T CD4+ (menos de 200 copias/milímetro cúbico (mm^3^)/igual o superior a 200 copias/mm^3^); eritrocitos (4,5 a 6,1 mm^3^/menos de 4,49 mm^3^), hemoglobina (menos de 12,79 miligramos (mg)/decilitros (dL)/12,8 a 17,8 mg/dL/17,9 mg/dL superior), hematócrito (inferior a 38,9 mg/dL/39 a 53 mg/dL/53,1 mg/dL superior), leucocitos (inferiores a 3599 mm^3^/3600 a 11000 mm^3^/11001 mm^3^ superiores), plaquetas (inferiores a 149.999 mm^3^/150.000 a 450.000 mm^3^/superiores 450.001 mm^3^) y glucosa en sangre (inferiores a 125 mg/dL y superiores a 126 mg/dL). Colesterol total (CT) (hasta 190 mg/dL/más de 191 mg/dL), lipoproteínas responsables del transporte del colesterol. Lipoproteínas de alta densidad (HDL) (menos de 39,1 mg/dL/40 a 60 mg/dL), lipoproteínas de baja densidad (LDL) (100 a 159 mg/dL/más de 160 mg/dL), triglicéridos (TG) (150 a 200 mg/dL/más de 201 mg/dL).

Los marcadores bioquímicos de electrolitos y función renal fueron urea (19,0 a 43,0 mg/dL/más de 43,1 mg/dL), creatinina (0,70 a 1,19 mg/dL/más de 1,20 mg/dL), sodio (137,0 a 145,9 mmol/L/más de 146 mmol/L), potasio (3,5 a 5,1 mmol/L/más de 5,2 mmol/L). Marcadores bioquímicos de la función hepática: aspartato aminotransferasa (AST) (hasta 40,9 U/L/superior a 41 U/L), alanina aminotransferasa (ALT) (hasta 41 U/L/superior a 41,1 U/L), bilirrubina total (BT) (0,3 a 1,20 mg/dL/más de 1,21 mg/dL), bilirrubina directa (BD) (hasta 0,20 mg/dL/más de 0,21), bilirrubina indirecta (BI) (hasta 1,0 mg/dL/más de 1,1 mg/dL).

### Recogida de datos

La recogida de datos se llevó a cabo entre febrero y junio de 2023 por el investigador principal del estudio y otro asistente de recolección, ambos debidamente formados.

Para controlar los sesgos, tanto en la medición como en la selección, los historiales médicos fueron analizados y recopilados por ambos investigadores, y luego transportados a una base de datos en hojas de cálculo Excel^®^ (Microsoft, versión 10, EE. UU.). Así, los datos pasaban por un proceso de doble entrada, seguido de validación para promover su fiabilidad. Cabe destacar que no fue necesario un tercer revisor para el análisis de los historiales médicos.

### Procesamiento y análisis de datos

En cuanto al análisis de datos, se utilizó el *software Statistical Package for the Social Sciences* (SPSS), versión 24. Se aplicaron técnicas estadísticas descriptivas y medidas de asociación. Las variables continuas se presentaron mediante sus medidas de tendencia central, es decir, media, desviación estándar (DE) (±), mínima y máxima. Las variables categóricas se expresaron como distribución de frecuencia absoluta (n) y relativa (%) de frecuencia. Además, para comparar los grupos según el resultado clínico, es decir, muerte o alta hospitalaria, se utilizaron pruebas no emparejadas, además de la prueba de chi-cuadrado de Pearson o la prueba exacta de Fisher, considerando la naturaleza de variables continuas y categóricas, respectivamente.

Para determinar el riesgo de muerte de los participantes, se utilizó el modelo de riesgos proporcionales de Cox, que se empleó para reconocer las covariables que influyeron en el tiempo desde el inicio del ingreso hospitalario hasta el resultado del caso. Cabe destacar que en el modelo de análisis multivariante se incluyeron variables con un valor p ≤0,05 en el análisis bivariante. El ajuste global del modelo se obtuvo mediante la prueba de razón de verosimilitud. Hubo una asociación en relación con los marcadores bioquímicos y el resultado (muerte y alta hospitalaria) y las covariables individuales asociadas a los resultados, con p<0,05.

Así, las covariables se incluyeron en el modelo de regresión de Cox para los análisis. Se estableció como un p-crítico <0,05 en el análisis múltiple para aceptar la asociación entre el resultado de la persona y las alteraciones bioquímicas. La razón de riesgo (HR) se calculó en los análisis de crudo y ajustados con un intervalo de confianza del 95%, y la calidad del ajuste se evaluó mediante la distribución de los residuos.

### Aspectos éticos

La investigación fue aprobada por el Comité de Ética en la Investigación (CEP) de la *Universidade Franciscana* (UFN) el 19/12/2022, bajo el dictamen n.º 5.828.312.

## Resultados

Durante el periodo del estudio, 1.328 personas fueron ingresadas en la unidad clínica, de las cuales 117 fueron hospitalizaciones relacionadas con la infección por VIH. Tras aplicar los criterios de inclusión y exclusión, se ignoraron 15 historiales médicos (10 por una estancia inferior a 72 horas y cinco por presentar más del 80,0% de los datos incompletos), lo que resultó en 102 participantes elegibles para el análisis.

Del total de la muestra, la edad media era de 43,3 años (SD±12), con una edad mínima de 18 años y un máximo de 78 años. En cuanto al resultado clínico, 27 (26,5%) casos murieron durante el estudio, causados por el VIH, y 75 (73,5%) fueron dados de alta del hospital. La duración media de la estancia hospitalaria registrada en los registros médicos de los participantes fue de 19 días, con un periodo mínimo de cinco y un máximo de 45 días (SD±9).

Del total, 60 (58,8%) eran hombres y, en relación con el uso de TAR, 75 (73,5%) estaban bajo tratamiento durante la hospitalización y 27 (16,8%) tenían interrupción. Además, las características clínicas y la prevalencia de comorbilidades presentadas por los participantes se detallan en la Tabla 1 según el resultado clínico.

**Tabla 1:** Características sociodemográficas y clínicas de las personas hospitalizadas con VIH. Santa Maria, RS, Brasil, 2023

Variable	Resultados clínicos				
	Alta hospitalaria		Muerte		
	n*	(%)^†^	n*	(%)^†^	Valor p^‡^
Género					**0,013** ^§^
Masculino	39	52,0%	22	81,5%	
Femenino	36	48,0%	5	18,5%	
Uso de la ART^||^					0,000^¶^
Sí	75	100%	-	-	
No	-	-	27	100%	
Diabetes Mellitus					**0,000** ^§^
Sí	22	29,3%	17	62,9%	
No	53	70,7%	10	37,1%	
Enfermedad renal crónica					**0,002** ^§^
Sí	69	92,0%	18	66,7%	
No	6	8,0%	9	33,3%	
Hipertensión arterial sistémica					**0,000** ^§^
Sí	25	33,3%	19	70,4%	
No	50	66,7%	8	29,6%	
Coinfección TB/VIH**					0,357
Sí	23	30,7%	11	40,7%	
No	52	69,3	16	59,3%	
Otras comorbilidades					**0,028** ^¶^
Sí	25	33,3%	4	14,8%	
No	50	66,7%	23	85,2%	

*n = Tamaño de la muestra según el resultado; ^†^% = Porcentaje; ^‡^valor p = Valor estadísticamente significativo en el nivel del 5% (p ≤ 0,05); ^§^Test de qui-cuadrado de Pearson; ^||^ART = Terapia Antirretroviral; ^¶^La prueba exacta de Fisher; **TB/VIH = Tuberculosis/Virus de la inmunodeficiencia humana

En cuanto a la razón de la admisión de los participantes en la unidad clínica de hospitalización, se identificaron las siguientes: inmunosupresión - 30 personas (29,4%), neumonía - 10 (9,8%), tuberculosis - seis (5,9%), infección oportunista en investigación - cuatro (3,9%), criptococcosis - tres (2,9%), desnutrición - tres (2,9%), hepatitis viral - tres (2,9%), herpes zóster - tres (2,9%), pancreatitis - tres (2,9%), sarcoma de Kaposi - tres (2,9%), abuso sexual - dos (2,0%), candidiasis oral y esofágica - dos (2,0%), neurotoxoplasmosis - dos (2,0%), neumocistosis - dos (2,0%), sepsis - dos (2,0%) y otros - 24 (23,5%).

La Tabla 2 presenta y compara las diferencias entre los marcadores hematológicos de los participantes del estudio y su relación con los resultados clínicos. Se observó una asociación estadística entre la muerte y los participantes que tenían un recuento de eritrocitos por debajo de 4,49/mm^3^, niveles de hemoglobina por debajo de 12,79 mg/dL y plaquetas por debajo de 149.999 mm^3^, con un valor p=0,000. 

**Tabla 2:** Asociación de biomarcadores hematológicos y perfil lipídico en personas hospitalizadas con VIH, según el resultado clínico. Santa Maria, RS, Brasil, 2023

Variable	Resultados clínicos				
	Alta hospitalaria		Muerte		
	n*	(%)^†^	n*	(%)^†^	valor p^‡^
Eritrocitos (mm^3§^)					**0,000** ^||^
4,5 a 6,1	51	50,0%	3	2,9%	
Inferior a 4,49	24	23,5%	24	23,6%	
Hemoglobina (mg/dL^¶^)					**0,000** ^||^
Inferior 12,79	21	20,7%	25	24,5%	
12,8 a 17,8	54	52,9%	2	2,0%	
Hematocrito (mg/dL^¶^)					0,321**
Inferior 38,9	67	65,7%	22	21,6%	
39 a 53	8	7,8%	5	4,9%	
Leucocitos (mm^3§^)					0,836**
Inferior 3599	11	10,8%	4	3,9%	
3600 a 11000	46	45,2%	15	14,7%	
Superior 11001	18	17,6%	8	7,8%	
Plaquetas (mm^3§^)					**0,000** ^||^
Inferior 149.999	11	10,8%	14	13,7%	
150.000 a 450.000	59	57,8%	10	9,8%	
Superior 450.001	5	4,9%	3	2,9%	
Colesterol Total (mg/dL^¶^)					**0,000** ^||^
Hasta 190	53	52,0%	3	2,9%	
Superior a 191	22	21,6%	24	23,5%	
HDL^††^ (mg/dL^¶^)					**0,000** ^||^
Inferior a 39,9	38	37,2%	26	25,5%	
40 a 60	37	36,3%	1	1,0%	
LDL^‡‡^ (mg/dL^¶^)					**0,000** ^||^
100 a 159	47	46,1%	3	2,9%	
Superior 160	28	27,5%	24	23,5%	
Triglicéridos (mg/dL^¶^)					**0,000** ^||^
150 a 200	64	61,7%	1	1,0%	
Superior a 201	11	10,8%	27	26,5%	

*n = Tamaño de la muestra según el resultado; ^†^% = Porcentaje; ^‡^p-valor = Valor estadísticamente significativo en el nivel del 5% (p ≤ 0,05); ^§^mm^3^ = Milímetros cúbicos; ^||^La prueba exacta de Fisher; ^¶^mg/dL = Miligramos por decilitro; **La prueba del qui-cuadrado de Pearson; ^††^HDL = Lipoproteínas de alta densidad; ^‡‡^LDL = Lipoproteína de baja densidad

Además, se encontró una asociación entre el perfil lipídico y el resultado clínico. Los participantes con colesterol total superior a 191 mg/dL, HDL inferior a 39,9 mg/dL, LDL superior a 160 mg/dL y triglicéridos superiores a 201 mg/dL mostraron una mayor asociación con la muerte (p=0,000).

En cuanto a los marcadores de función renal de los participantes, se identificó que existe una asociación estadística entre el resultado de la muerte y los marcadores de urea, creatinina, sodio y potasio, como se destaca en la Tabla 3.

**Tabla 3:** Asociación de biomarcadores renales en personas hospitalizadas con VIH, según el resultado clínico. Santa Maria, RS, Brasil, 2023

Variable	Resultados clínicos				
	Alta hospitalaria		Muerte		
	n*	(%)^†^	n*	(%)^†^	p-valor^‡^
Urea (mg/dL^§^)					**0,000** ^||^
9,0 a 43,0	8	7,8%	20	19,6%	
Superior a 43,1	67	65,7%	7	6,9%	
Creatinina (mg/dL^§^)					**0,000** ^¶^
0,70 a 1,19	62	60,8%	4	3,9%	
Superior a 1,20	13	12,7%	23	22,6%	
Sodio (mmol/L**)					**0,000** ^||^
137,0 a 145,9	69	67,6%	10	9,8%	
Superior a 146	6	5,9%	17	16,7%	
Potasio (mmol/L^**^)					**0,000** ^¶^
3,5 a 5,1	71	69,6%	6	5,9%	
Superior a 5,2	4	3,9%	21	20,6%	

*n = Tamaño de la muestra según el resultado; ^†^% = Porcentaje; ^‡^valor p = Valor estadísticamente significativo en el nivel del 5% (p ≤ 0,05); ^§^mg/dL = Miligramos por decilitro; ^||^La prueba del qui-cuadrado de Pearson; ^¶^La prueba exacta de Fisher; **mmol/L = Milimol por litro

Como se muestra en la Tabla 4, la evolución hasta la muerte mostró una asociación significativa con marcadores hepáticos. El grupo de muerte tuvo una prevalencia mayor de resultados que indican un pronóstico peor, específicamente para AST (p=0,000), ALT (p=0,001), BT (p=0,003) y BD (p=0,032).

**Tabla 4:** Asociación de biomarcadores hepáticos en personas hospitalizadas con VIH, según el resultado clínico. Santa Maria, RS, Brasil, 2023

Variable	Resultados clínicos				
	Alta hospitalaria		Muerte		
	n*	(%)^†^	N*	(%)^†^	valor p^‡^
AST^§^ (U/L^||^)					**0,000** ^¶^
Hasta 40,9	47	46,1%	4	3,9%	
Superior a 41	28	27,5%	23	22,5%	
ALT** (U/L^||^)					**0,001** ^††^
Hasta 41	60	58,8%	10	9,8%	
Superior 41,1	15	14,7%	17	16,7%	
Bilirrubina total (mg/dL^‡‡^)					**0,003** ^††^
0,3 a 1,20	41	40,2%	5	4,9%	
Superior 1,21	34	33,3%	22	21,6%	
Bilirrubina directa (mg/dL^‡‡^)					0,052^††^
Hasta 0,20	45	44,1%	12	11,8%	
Superior 0,21	30	29,4%	15	14,7%	
Bilirrubina indirecta (mg/dL^‡‡^)					**0,032** ^¶^
Hasta 1,0	22	21,6%	2	2,0%	
Superior a 1,1	53	52,0%	25	24,5%	

*n = Tamaño de la muestra según el resultado; ^†^% = Porcentaje; ^‡^valor p = Valor estadísticamente significativo en el nivel del 5% (p ≤ 0,05); ^§^AST = Aspartate aminotransferasa; ^||^U/L = Unidades por litro; ^¶^La prueba exacta de Fisher; **ALT = Alanina aminotransferasa; ^††^Prueba del chi cuadrado de Pearson; ^‡‡^mg/dL = Miligramos por decilitro

Según los datos expresados en la Tabla 5, el modelo ajustado identificó una asociación con la muerte entre personas que tenían VC igual o superior a 1000 copias/mL (p=0,023), linfocitos T CD4 iguales o inferiores a 200 células/mm^3^ (p=0,035), glucosa en sangre igual o mayor a 126 mg/dL (p=0,017), creatinina igual o mayor a 1,20 mg/dL (p=0,012) y triglicéridos iguales o superiores a 201 mg/dL (p=0,000).

**Tabla 5:** Análisis de regresión de Cox bruta y ajustada para el riesgo de muerte en personas hospitalizadas con VIH. Santa Maria, RS, Brasil, 2023

Variable	HR* bruto (IC 95%^†^)	p^‡^	HR* ajustado (IC 95%^†^)	p^‡^
Carga viral (copias/mL)				
Igual ou superior a 1000	9,78 (3,23-12,43)	0,000	3,99 (1,25-12,85)	**0,023** ^§^
L T-CD4+^||^ (copias/mm^3¶^)				
Igual o inferior a 200 células/mm^3^	4,12 (1,78-2,12)	0,008	1,92 (1,85-2,36)	**0,035** ^§^
Glucosa (mg/dL**)				
Superior a 126	4,49 (2,29-8,36)	0,005	5,40 (2,11-8,21)	**0,017** ^§^
Urea (mg/dL**)				
Superior a 43,1	0,16 (0,06-1,26)	0,007	0,19 (0,09-1,99)	0,195
Creatinina (mg/dL**)				
Superior a 1,20	10,12 (5,23-15,36)	0,009	9,75 (2,56-14,98)	**0,012** ^§^
Sodio (mmol/L^††^)				
Superior a 146	2,19 (1,34-3,60)	0,009	2,12 (1,27-3,51)	0,051
Potasio (mmol/L^††^)				
Superior a 5,2	3,25 (0,81-18,95)	0,007	5,23 (0,85-45,69)	0,089
Colesterol Total (mg/dL**)				
Superior a 191	0,52 (0,08-2,36)	0,001	0,23 (0,23-2,15)	0,212
HDL^‡‡^ (mg/dL**)				
Inferior a 39,9	2,45 (0,88-6,33)	0,008	0,68 (0,23-1,65)	0,654
LDL^§§^ (mg/dL**)				
Superior a 160	2,45 (0,85-5,71)	0,004	1,63 (0,63-4,71)	0,854
Triglicéridos (mg/dL**)				
Superior a 201	12,15 (2,75-15,63)	0,008	11,75 (4,18-23,26)	**0,000** ^§^

*HR = *Hazard Ratio* (Relación de Riesgo); ^†^IC = Intervalo de confianza del 95%; ^‡^p = Nivel de significancia; ^§^Resultado estadísticamente significativo; ^||^L T CD4+ = Linfocitos T-CD4+; ^¶^mm^3^ = Milímetros cúbicos; **mg/dL = Miligramos por decilitros; ^††^mmol/L = Milimol por litro; ^‡‡^HDL = Lipoproteínas de alta densidad; ^§§^LDL = Lipoproteína de baja densidad

Además, se observa que los participantes con resultados de VC superiores a 1000 copias/mL tenían aproximadamente 3,99 veces más riesgo de progresar a la muerte, en comparación con aquellos con resultados inferiores a 1000 copias/mL (frecuencia cardíaca ajustada: 3,99; IC 95%: 1,25-12,85). Las personas con un recuento de linfocitos T CD4+ inferior a 200 células/mm3 tenían un riesgo de muerte 1,92 veces mayor en comparación con aquellas que tenían un recuento de 200 mm^3^ o más (HR ajustado: 1,92; IC 95%: 1,85-2,36).

Los participantes que obtuvieron un resultado superior a 126 mg/dL en el biomarcador de glucosa en sangre tuvieron un riesgo 5,40 veces mayor de morir durante la hospitalización, en comparación con quienes tenían un valor inferior a 125 mg/dL (HR ajustado: 5,40; IC 95%: 2,11-8,21). Se observó que los participantes con un valor marcador de creatinina superior a 1,20 mg/dL tenían un riesgo de muerte 9,75 veces mayor, en comparación con aquellos con valores inferiores a 1,19 mg/dL (HR ajustado: 9,75; IC 95%: 2,56-14-98).

Además, los participantes que tenían niveles de triglicéridos superiores a 201 mg/dL tenían un riesgo de muerte 11,75 veces mayor que aquellos con valores más bajos (HR ajustado: 11,75; IC 95%: 4,18-23,26).

## Discusión

Para analizar las alteraciones entre los biomarcadores presentados por personas hospitalizadas con infección por VIH, se observa que los valores de CV refuerzan lo que ya se ha identificado en la literatura científica. Los estudios indican que las personas que viven con VIH y utilizan TAR, con una carga viral indetectable o entre 200 y 1000 copias/mL, tienen un mejor pronóstico de infección^20^. Por otro lado, las personas hospitalizadas con una CV superior a 1000 copias/mL están directamente asociadas con marcadores avanzados de SIDA y tienen un mayor riesgo de progresión hacia la muerte^21^.

En este contexto, el presente estudio refuerza que las personas que viven con VIH y que tienen recuentos de linfocitos T-CD4+ inferiores a 200 copias/mm³ son más susceptibles a infecciones oportunistas y, en consecuencia, tienen una mayor probabilidad de progresión hacia la muerte debido a la enfermedad. Un estudio brasileño que analizó el perfil de mortalidad de 109.636 personas que vivían con VIH entre 2014 y 2019 identificó resultados similares a los hallazgos del presente estudio, mostrando que tasas más bajas de linfocitos T-CD4+ estaban asociadas con la mortalidad^22^. Además, los resultados de este estudio demuestran que las personas con recuentos de linfocitos T-CD4+ inferiores a 200 copias/mm^3^ tienen aproximadamente 1,92 veces más riesgo de morir en comparación con aquellas con valores iguales o superiores a 200 mm³ (p=0,035).

De manera similar, un estudio realizado en Tanzania identificó que las personas que viven con VIH tienen un riesgo de desarrollar DM hasta cinco veces mayor que quienes no viven con el virus^23^. Esta asociación se vuelve aún más significativa al considerar los hallazgos del presente estudio, en el que los participantes con niveles de glucosa en sangre superiores a 126 mg/dL tenían un riesgo 5,4 veces mayor de morir. Cabe destacar que el aumento de los niveles de glucosa en sangre en personas que viven con VIH puede explicarse, en gran medida, por la resistencia a la insulina inducida por la TAR, un factor que contribuye al desarrollo de la DM^23^.

Además, cabe destacar que poco después de la introducción de la TAR, las personas que viven con VIH experimentan reconstitución inmunitaria, un proceso que puede resultar en un aumento de la secreción de factores de necrosis tumoral alfa (TNF-α). Este fenómeno puede inducir resistencia a la insulina a través de varios mecanismos que implican la cascada de señalización de los receptores de insulina^24^. En vista de ello, es esencial que los profesionales sanitarios mantengan un compromiso continuo con estrategias preventivas, incluyendo el seguimiento regular de los niveles glucémicos. Este enfoque pretende reducir la progresión hacia la DM, evitando la doble carga de la enfermedad en esta población y, en consecuencia, reduciendo las probabilidades de un pronóstico peor^23^.

El análisis de la asociación entre los marcadores renales y el resultado de la muerte reveló una asociación significativa entre los marcadores renales y el riesgo de muerte. Las personas con valores superiores a 1,20 mg/dL tenían un riesgo de muerte 9,75 veces mayor. En vista de esto, es esencial enfatizar que la insuficiencia renal representa un problema grave en las personas que viven con VIH, asociándose a varias causas, incluyendo nefropatías derivadas del VIH, nefrotoxicidad por medicamentos, procedimientos médicos y, especialmente, enfermedad renal crónica, que puede ser causada o agravada por comorbilidades^25^ ^)-(^ ^26^.

Un estudio realizado en Estados Unidos indicó que las personas que viven con VIH tienen un riesgo de desarrollar insuficiencia renal hasta 13 veces mayor que quienes no viven con el virus, un valor superior al identificado en el presente estudio^27^. Esta divergencia podría estar relacionada con el acceso libre y universal al tratamiento en Brasil, lo que podría contribuir a un mejor control de las complicaciones renales en esta población.

Además, el estudio sugiere que todas las personas diagnosticadas con VIH deben someterse a una evaluación detallada de alteraciones renales, considerando la posibilidad de coexistencia de múltiples factores desencadenantes^27^. Esta recomendación está en línea con los protocolos nacionales, que buscan garantizar un seguimiento adecuado de las complicaciones asociadas al VIH^28^.

Los marcadores del perfil lipídico resultaron relevantes en el estudio, ya que se asociaron con el resultado de la muerte. En Estados Unidos, un estudio realizado con 981 personas con VIH identificó resultados similares, mostrando que el 41% de los participantes presentaba niveles altos de CT (p=0,000) y TG, mientras que el 21,4% de las personas que fallecieron tenían asociación con estos marcadores (p=0,002)^4^. Por tanto, se observa que el perfil lipídico encontrado en este estudio presenta valores superiores a los del estudio mencionado, pero mantiene la misma asociación significativa con la muerte como resultado (p=0,000). 

Un estudio brasileño^22^ advierte sobre la importancia de que los profesionales de la salud consideren estrategias orientadas al autocuidado y la adopción de hábitos de vida saludables, con el objetivo de minimizar estos factores de riesgo. Estos factores están directamente relacionados con complicaciones cardiovasculares, que representan la principal causa de morbilidad y mortalidad entre las personas que viven con VIH^29^.

Además, la alta prevalencia de comorbilidades en la muestra del presente estudio, con un 38,2% de diagnósticos de DM, 41,2% de HAS y 85,3% de DRC, está en línea con lo observado a nivel mundial en personas que viven con VIH. La evolución en la prescripción de medicamentos para enfermedades crónicas y la creciente prevalencia de estas comorbilidades entre estas personas, como demuestra un estudio reciente en Bélgica, subrayan la necesidad de un seguimiento continuo y un manejo adecuado para evitar resultados desfavorables^30^. La presencia de estas comorbilidades, por sí sola, ya es un factor de peor pronóstico, y su interacción con las alteraciones bioquímicas discutidas en este estudio puede intensificar el riesgo de muerte.

A la luz de los hallazgos de este estudio, corroborados por la literatura científica^4^ ^),(^ ^29^, se destaca la importancia de adherirse a la TAR y la necesidad de desarrollar hábitos de vida saludables para minimizar los resultados clínicos desfavorables. Aunque la TAR se asocia con un mayor riesgo de alteraciones metabólicas y una mayor probabilidad de desarrollar enfermedades cardiovasculares a largo plazo, sigue siendo la estrategia más eficaz para el manejo de la infección por VIH, proporcionando un mejor pronóstico y una mejor calidad de vida a las personas que viven con VIH^31^.

La prevalencia de enfermedades hepáticas en personas con VIH varía entre el 4% y el 18% en distintas regiones del mundo, y las tasas de mortalidad relacionadas con daños hepáticos son hasta 10 veces más frecuentes en esta población en comparación con la población general^32^. Un estudio realizado en España confirmó este hallazgo, confirmando que los participantes que vivían con VIH y tenían insuficiencia hepática tenían un pronóstico peor en comparación con aquellos sin infección por VIH. Las principales causas de enfermedad hepática en esta población incluían coinfecciones con el virus de la hepatitis C (VHC) y el virus de la hepatitis B (VHB), lesiones hepáticas inducidas por fármacos, enfermedades alcohólicas y enfermedad hepática grasa no alcohólica^33^.

Los autores también subrayan la importancia del compromiso de los profesionales sanitarios, desde la fase inicial del diagnóstico de la infección hasta el seguimiento de los marcadores hepáticos y lipídicos, independientemente de la combinación de TAR utilizada^33^. Este seguimiento es esencial para reducir el riesgo de progresión de la enfermedad hepática y el desarrollo de carcinoma hepatocelular.

En vista de los aspectos destacados, se destaca la relevancia de la práctica clínica y la investigación futura sobre el tema, ya que la interrupción del tratamiento, la recurrencia y la coinfección por VHC en personas con VIH requieren un seguimiento riguroso, especialmente en lo que respecta a la fibrosis residual. Además, estudios bien diseñados que investigan el desarrollo de trastornos metabólicos a largo plazo en personas coinfectadas pueden contribuir significativamente a reducir la magnitud de este problema, ayudando al desarrollo de estrategias terapéuticas más eficaces^33^.

El análisis de las causas de hospitalización de los participantes, que incluyeron infecciones oportunistas como neumonía (9,9%), tuberculosis (5,9%), criptococcosis (2,9%) y sepsis (2,0%), refuerza la importancia de prestar atención a la inmunosupresión severa. La diversidad de estas condiciones clínicas subraya la vulnerabilidad de esta población a complicaciones graves, lo que podría explicar la fuerte asociación entre biomarcadores alterados y el resultado de la muerte^8^ ^)-(^ ^9^.

Además del análisis de los marcadores que permanecieron en el modelo de regresión, este estudio también investigó la relevancia de los biomarcadores hematológicos. Aunque no tenían el poder estadístico para permanecer en el modelo, estos biomarcadores son de gran importancia para la inmunidad de las personas que viven con VIH. El análisis de los resultados de los hemogramas de los participantes reveló una asociación significativa entre la presencia de anemia y la progresión hacia la muerte p=0,000. Este hallazgo refuerza los resultados de un metaanálisis, que identificó la presencia de anemia en el 78,8% de las personas con VIH, asociando esta condición con un peor pronóstico de salud^34^.

Por ello, se destaca la importancia de realizar exámenes periódicos en personas con VIH, así como la atención de los profesionales sanitarios a sus alteraciones, ya que la anemia se ha citado como un marcador de peor pronóstico de infección y se asocia principalmente con una reducción de la supervivencia en estas personas^34^. Cabe destacar que varios factores pueden influir en el desarrollo de la anemia en personas que viven con VIH, incluyendo el uso de TAR, la presencia de mediadores inflamatorios y las coinfecciones asociadas a la infección. Estos factores pueden comprometer la proliferación y diferenciación de células madre hematopoyéticas/progenitoras durante la hematopoyesis, culminando en el cuadro clínico de anemia^35^.

En cuanto a las limitaciones del estudio, se destaca el uso de fuentes secundarias, que pueden generar sesgos como la falta de información, así como la ausencia de datos importantes en el sistema de registro. Otro aspecto que requiere atención es el hecho de que el estudio no consideró los medicamentos utilizados por los participantes durante el periodo de hospitalización. Aunque el estudio se realizó en un solo hospital en el interior del estado de Rio Grande do Sul, los hallazgos están en línea con la literatura nacional e internacional, lo que refuerza su validez. Sin embargo, al interpretar estos resultados, se recomienda precaución debido a las particularidades del contexto en el que se llevó a cabo la investigación.

Este estudio busca contribuir con un marco teórico y práctico relevante para el área de enfermería, ya que los resultados de la investigación demostraron la relevancia de la evaluación clínica de biomarcadores por parte de profesionales sanitarios como predictores de un peor pronóstico, contribuyendo así a la planificación de la atención. Además, los hallazgos ayudan en la toma de decisiones clínicas de las personas que viven con VIH, ya que identificaron asociaciones sólidas entre las alteraciones bioquímicas presentadas y la ocurrencia de la muerte. Se recomiendan acciones que prioricen el diagnóstico precoz, la solicitud inmediata de pruebas bioquímicas y el seguimiento de estos marcadores durante todos los ciclos de seguimiento de personas que viven con VIH.

## Conclusión

Se encontró que las personas hospitalizadas con VIH con VC superior a 1000 copias/mL y un recuento bajo de linfocitos T-CD4+ (<200 copias/mm³) estaban asociadas con el resultado clínico de la muerte, reforzando así la importancia del control virológico e inmunológico en el manejo clínico de la infección por VIH.

Otro hallazgo relevante fue la asociación del resultado clínico de la muerte con biomarcadores metabólicos y renales, como niveles de glucosa en sangre superiores a 126 mg/dL y niveles altos de creatinina superiores a 1,20 mg/dL. Finalmente, los participantes con niveles de triglicéridos superiores a 201 mg/dL representaron el mayor factor de riesgo identificado en este estudio, reforzando la necesidad de un seguimiento estricto de este parámetro en esta población.

Así, según los hallazgos del estudio, se concluye que los biomarcadores inmunológicos, metabólicos, renales y lipídicos desempeñan un papel crucial en la evaluación pronóstica de personas hospitalizadas con VIH. Por tanto, la identificación temprana de estas alteraciones es esencial para ayudar en la implementación de estrategias terapéuticas más efectivas, con el objetivo de reducir la mortalidad y mejorar el manejo clínico de estas personas.

## Data Availability

Todos los datos generados o analizados durante este estudio están incluidos en este artículo publicado.
